# Prediction of the Co-receptor usage of the main worldwide HIV-1 subtypes, CRF, and CRF35-AD in Iranian patients via the five genotypic tools

**DOI:** 10.1016/j.bbrep.2025.101939

**Published:** 2025-02-17

**Authors:** Ava Hashempour, Shokufeh Akbarinia, Nastaran Khodadad, Farimah Safari, Zeinab Mehrabi

**Affiliations:** a HIV/AIDS Research Center, Institute of Health, Shiraz University of Medical Sciences, Shiraz, Iran; b Department of Internal Medicine, Shiraz University of Medical Sciences, Shiraz, Iran

**Keywords:** Co-receptor, *HIV-1*, *V3* tropism, Major subtypes, CRF35-AD

## Abstract

HIV-1 has various subtypes and CRFs, each with unique genetic attributes that impact the virus's spread, disease development, and response to treatment in different populations. Determining V3 tropism is crucial for utilizing CCR5 antagonists and understanding why certain HIV-1 subtypes are more pathogenic than others are. Genotypic coreceptor usage of 603 major subtypes of A, B, C, AE, and CRF35-AD is investigated via five bioinformatics tools (PhenoSeq, WebPSSM, Geno2Pheno, Net charge, and the 11/25 rule). This study examined crown motifs, N-glycosylation sites, and T8V mutations in all subtypes. R5 viruses are common in subtypes A, B, C, and CRF35-AD. These data indicate that R5 viruses in subtypes A and B are more prone to crown motif formation. The first report assessed the tropism of common HIV-1 subtypes and reported that CCR5 inhibitors could help treat patients with all subtypes but not AE. WebPSSM is a suitable method for determining HIV-1 tropism in different subtypes. Finally, large cohorts to assess virological response to CCR5 inhibitors would provide deep insight into the practicality of genotypic methods in clinical settings.

## Highlights

• R5 viruses are highly prevalent in A, B, and C subtypes.
• WebPSSM is a suitable method to determine *HIV* tropism in A, B, C, AE, and CRF35-AD subtypes.
• R5 viruses in subtypes A and B are more prone to crown motif formation.

## 1 Introduction

New viral diseases and outbreaks of deadly diseases pose significant public health risks in both developing and developed countries. This is an important concern because these infectious diseases can spread rapidly and have devastating consequences for individuals, communities, and entire populations. Among the various infectious diseases, HIV-1 infection remains a major global concern [[1], [2], [3], [4], [5]].

There were almost 39 million *HIV-1*-infected patients in the world in 2022, 1.5 million of whom were children (<15 years old) and 37.5 million adults [6,7]. However, improvement in antiretroviral treatment (ART) increased life expectancy in patients infected with *HIV-1* [8,9]. In contrast, the error-prone viral replication process produces high genetic diversity that leads to immune system evasion, undermines treatment efficacy, and promotes disease progression [10,11].

Many host and viral factors are linked with *HIV-1* pathogenesis and disease outcomes, namely, *HIV-1* subtypes, coinfection with other pathogens, stage of the disease, different types of posttranslational modifications [12,13], cytokine and chemokine levels [14]. Another viral factor associated with *HIV-1* disease progression is the tropism of the virus toward the various immune cells [15,16].

The definition of tropism in *HIV-1* is the ability of the virus to enter specific cells to complete viral replication to facilitate productive infection. Tropism influences the extent of the cytopathic effect of *HIV-1* viruses on peripheral blood mononuclear cells (PBMC) [17]. The tropism feature of *HIV-1* limits the cell population and hosts that can be infected with this virus; for example, *HIV-1* cannot infect all of human cells including erythrocytes and neurons as well as the animals such as mice, rats, and primates [18].

The *HIV-1-V3* loop that is located in the envelope protein plays a pivotal role in the determination of the coreceptor usage or tropism [19]. Various factors define the *HIV-1* tropism: protein sequences outside the *gp120* regions, several HIV genes, including Vpr, Tat, and LTR region [20] and different features of the *V3* loop, namely, positions of N-linked glycosylation sites, sequence Net charge, and mutation in the amino acid at the position of 34 [21].

*HIV-1* tissue tropism affects viral pathogenicity; for example, *HIV-*1 that uses the CCR5 receptor for entry fails to induce syncytium, while *HIV-1* with CXCR4 tropism can propagate rapidly, lead to syncytium formation and cell death [22]. Identification of *HIV-1* tropism is not only critical for the specific prescription of coreceptor antagonists in *HIV-1*-infected patients but also provides an understanding of why some subtypes of *HIV-1*, such as AE, that predominantly are X4 tropism, are more virulent and lead to advanced stages of the disease [[23], [24], [25]].

Based on the *HIV-1* tropism, drugs have been developed against the CCR5 co-receptor Maraviroc (MVC) is a CCR5-blocking drug that was approved for the treatment of HIV-1 infection [26], and Vicriviroc, which binds to CCR5 of human cells and inhibits the entry of HIV-1 into CD4 cells was never approved by the US FDA [27]. Various algorithms were designed to predict *HIV-1* tropism, including Net charge rule, 11/25 rule, Geno2Pheno, WebPSSM, and PhenoSeq.

In this study, we evaluated the tropism of 480 isolates from Group M subtypes (A, B, C, and AE) and 123 subtype CRF35-AD isolates obtained from Iranian patients via five genotypic bioinformatics methods (PhenoSeq, Geno2Pheno, WebPSSM, Net charge, and the 11/25 rule). In addition, the effects of the T8V mutation, crown motif, and N-glycosylation on virus tropism were investigated. Additionally, this is the first study to focus on the main subtypes concurrently. Cutting-edge versions of tropism prediction software were employed in the analysis. Previous studies relied on the complete gp120 sequence for predicting tropism, whereas our research focused only on the V3 sequence for more precise results.

## 2 Materials and methods

### 2.1 Retrieval of *HIV-1-V3* sequence data for major subtypes A, B, C, and AE

To compare the *HIV-1* tropism of the four major subtypes (A, B, C, and AE), 480 of the full *HIV-1* genomes of the most recently submitted sequences in the Los Alamos National Laboratory (LANL) *HIV* Sequence Database (https://www.HIV.lanl.gov/content/index) were extracted.

### 2.2 Study population, RT-PCR, and sequencing

We compared the HIV-1 tropism of isolates from Iranian patients, as determined in a previous study [28], with that of the major subtypes. The samples from 123 patients were tested to determine the co-receptor usage along with other experimental and bioinformatic analyses. RNA extraction and nested PCR were performed with specific primers to amplify the *V3* region, followed by sequencing.

### 2.3 Coreceptor usage prediction

**Fig. 1:**
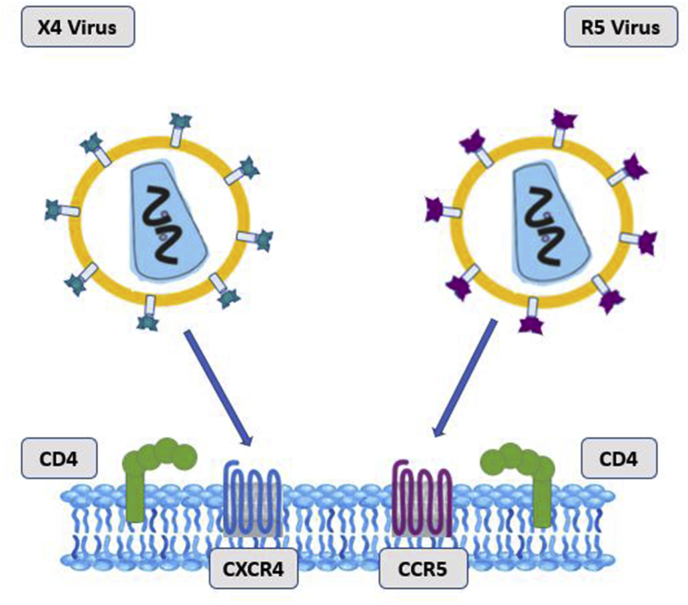
*V3/HIV-1* tropism and binding to coreceptors in host cells.

In this study, five bioinformatic approaches, including Geno2Pheno (https://coreceptor.geno2pheno.org/), PhenoSeq (https://tools.burnet.edu.au/phenoseq/), Net charge (https://pepcalc.com/), WebPSSM (https://indra.mullins.microbiol.washington.edu/webpssm/), and the 11/25 rule, were applied to evaluate *HIV-1* coreceptor usage for subtypes A, B, C, and recombinant forms of AE and 35-AD. The net charge rule is a simple method that calculates the net charge of the V3 sequence by subtracting the charged aspartic and glutamic acid residues [D + E] residues from the charged lysine and arginine residues [K + R]. A net charge of <+5 is associated with R5 viruses, whereas a net charge of ≥+5 is associated with X4 viruses [29], which interact with the CCR5 and CXCR4 coreceptors, respectively [30]. The 11/25 rule is another genotypic method that predicts *V3* tropism on the basis of the presence of basic amino acids, lysine (K) or arginine (R), at 11 or 25 positions of the *V3* sequence. In the *V3* sequence of the X4 strain, the amino acids at 11 or 25 positions are acidic, whereas in R5 viruses, such amino acids are basic [29]. In the 11/25 rule, the presence of positive amino acids charged at positions 11 or 25 in the *V3* sequences was defined through an alignment of all *V3* sequences by CLC Main Workbench software (CLC bio, Boston, MA) [10,[31], [32], [33]]. This rule is appropriate in clinical settings since the sensitivity of this rule is low for the detection of CXCR4-tropic viruses [30] and the conservative prescription of CCR5 antagonists. Among different genotypic tools, Geno2Pheno is a common engine based on the submission of *HIV-1-V3* sequences, with false positive rates (FPRs) of 5 % and 15 % (co-receptor). The false positive rate is a statistical metric that reflects the percentage of negative instances mistakenly identified as positive [34]. The other genotypic method is WebPSSM, which enables the examination of both nucleotide sequences and the amino acids of the *V3* region. The outcome of this tool illustrates the amino acids at locations 11 and 25. Combined with the results of the 11/25 rule, the number of amino acids in the *V3* loop with a positive charge and its net charge defines the tropism of X4 or R5 viruses [35]. PhenoSeq measures coreceptor usage with 5 % as the consequence level (cutoff) of the FPR via nucleotide or amino acid sequences of the *V3* region [36]. A schematic of V3/HIV-1 tropism and binding to coreceptors in host cells is shown in Fig. 1.

### 2.4 Determination of *V3*-loop mutations

The *V3* sequences obtained from the 123 amplified samples and all the retrieved sequences from the database were assembled into the related reference sequences (GenBank accession numbers: AY371159.1, M17451.1, U46016.1, U54771.1, and AB703607 for subtypes A, B, C, AE, and CRF35-AD, respectively) via the CLC sequence viewer.

### 2.5 Effects of the N-linked glycosylation site, T8V mutation, and crown motif on *HIV-1* tropism

Here, the nucleotide sequences of the *V3* regions of all the subtypes were translated and aligned with related reference sequences via MEGA10 [37] to identify the T8V mutation and crown motif (aa 15–18 of *V3*), which is called GPGQ and affects tropism and glycosylation. Moreover, the N-glycosylation of amino acids at positions 6 and 7 in the motif NTS and NNT of all *V3* sequences was defined via the online NetNGlyc server (http://www.cbs.dtu.dk/services/NetNGlyc/), with a threshold of 0.5 [12,38,39].

### 2.6 Statistics

**Fig. 2:**
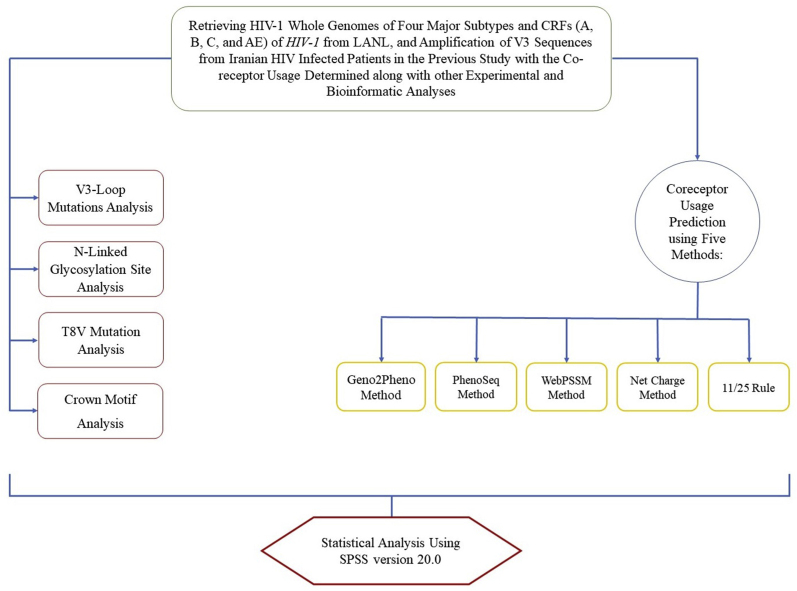
Overview of the flowchart of *V3* tropism prediction of *HIV-1* subtypes in this study.

Statistical analysis was performed via SPSS version 20.0 [40], and P values less than 0.05 were regarded as statistically significant. Nonparametric data were analyzed via median (Q1 and Q3), frequent (%), Kruskal‒Wallis, and Mann‒Whitney tests. The relationships between quantitative variables were explored via chi-squared and Spearman coefficient correlation tests. The flowchart of co-receptor usage prediction for HIV-1 subtypes is illustrated in Fig. 2.

## 3 Results

### 3.1 Tropism prediction in different subtypes using five genotypic methods

**Fig. 3:**
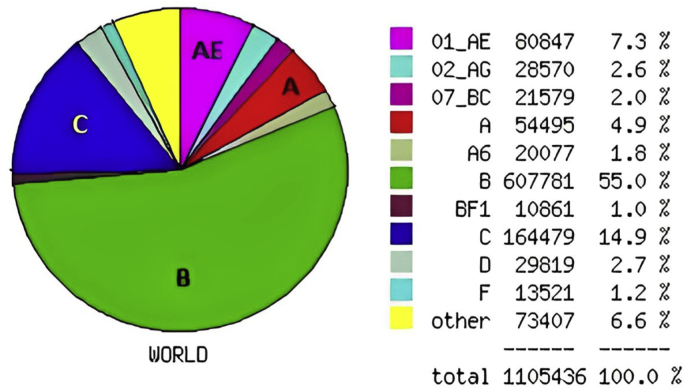
*HIV-1* subtypes and CRF distributions with the highest worldwide frequencies of A, B, C, and AE. The figure was adopted from the LANL database.

**Fig. 4:**
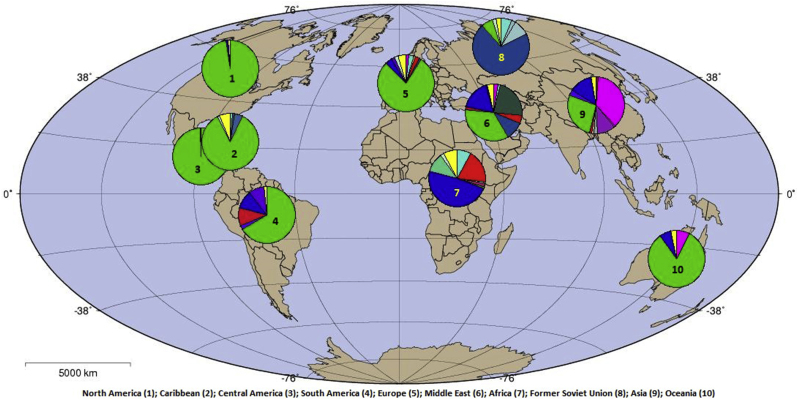
Geographic distribution of *HIV-1* subtypes and CRFs on the basis of the country of sequence isolation. The illustration was adapted from the LANL database.

On the basis of the *HIV-1* subtype distribution worldwide in the *HIV* Database up to date of 2/8/2023, the most frequent subtypes submitted in LANL were B, C, AE, and A, with percentages of 55 %, 14.9 %, 7.3 %, and 4.9 %, respectively (Fig. 3). In the LANL database, the *HIV-1* geography tool provides geographic distributions of *HIV-1-1* subtypes taken from various publications; therefore, there is no epidemiological framework. The geographic distribution of HIV-1 according to the country in which the sequence was isolated is shown on the maps (Fig. 4).

The gold standard for defining *HIV-1* tropism is the Trofile assay, which is categorized as a phenotypic method [41]. According to previous studies, the 11/25 and net charge rules had specificity and sensitivity percentages of 77 and 96, respectively. PhenoSeq, Geno2Pheno, and WebPSSM bioinformatic software presented specificity values of 80.3 %, 87 %, and 94 %, respectively, and sensitivity values of 78.4 %, 77 %, and 88 %, respectively [[42], [43], [44]].

Since the results of the phenotypic assay were not available for the studied sequences, five genotypic methods versus single testing were used for coreceptor usage prediction. In accordance with Symons’ findings, *V3* sequences with tropism were classified as X4 viruses [45]. The recommended methods for predicting the tropism of V3 in five subtypes are as follows: A (PhenoSeq and Net Charge), B, C, and AE (WebPSSM), and CRF35-AD (quintuplicate result).

As shown in Table 1 and **Additional** Table 1 (a-e), coreceptor usage was defined for 123 patient samples and 480 extracted sequences from the LANL *HIV* database. According to the tropism prediction procedure, the average X4 frequency in 603 sequences varied from 5.46 % (Geno2Pheno) to 14.48 % (PSSM), 20.94 % (PhenoSeq), 15.24 % (11/25 rule) and 32.16 % (Net Charge) (Table 2).

**Table 1:** Frequency of V3 tropism in subtypes A, B, C, AE and CRF-35 AD.

Subtypes	WebPSSM	PhenoSeq	Net charge	Geno2pheno	11/25 rule	Quintuplicate Results
**Iranian samples (CRF-35AD)**	R5: 121 (98.4 %) X4: 2 (1.6 %)	R5:110 (89.4 %) X4:13(10.6 %)	R5: 108 (87.8 %) X4: 2 (1.6 %) UD: 13 (10.6 %)	R5: 113 (91.9 %) X4: 10 (8.1 %)	R5:122 (99.2 %) X4: 1 (0.8 %)	R5: 103 (83.7 %) X4: 20 (16.3 %)
**A**	R5:114 (95 %) X4:6 (5 %)	R5:107 (89.2 %) X4:13 (10.8 %)	R5: 73 (60.8 %) X4: 43 (39.2 %)	R5: 120(100 %)	R5: 83 (69.2 %) X4:37 (30.8 %)	R5: 57 (47.5 %) X4: 63 (52.5 %)
**B**	R5:100 (83.3 %) X4:20 (16.7 %)	R5:53 (44.2 %) X4:67 (55.8 %)	R5: 41 (34.2 %) X4: 79 (65.8 %)	R5: 111 (92.5 %) X4: 9 (7.5 %)	R5:104 (86.7 %) X4:16 (13.3 %)	R5: 25 (20.8 %) X4: 95 (79.2 %)
C	R5:113 (94.2 %) X4:7 (5.8 %)	R5:109 (90.8 %) X4:11 (9.2 %)	R5: 85 (70.8 %) X4:35 (29.2 %)	R5: 118 (98.3 %) X4: 2 (1.7 %)	R5:119 (99.2 %) X4:1 (0.8 %)	R5: 78 (65 %) X4: 42 (35 %)
**AE**	R5:68 (56.7 %) X4:52 (43.3 %)	R5: 98 (81.7 %) X4: 22 (18.3 %)	R5: 90 (75 %) X4: 30 (25 %)	R5: 108 (90 %) X4: 12 (10 %)	R5: 102 (85 %) X4: 18 (15 %)	R5: 41 (34.2 %) X4: 79 (65.8 %)

**Table 2:** Frequency X4-rate tropism in 5 procedures.

X4 frequency (%) in subtype	Procedures				
	WebPSSM	PhenoSeq	Net charge	Geno2Pheno	11/25 rule
**CRF-35 AD subtype (Iranian samples)**	1.6	10.6	1.6	8.1	16.3
**A subtype**	5	10.8	39.2	0	30.8
**B subtype**	16.7	55.8	65.8	7.5	13.3
**C subtype**	5.8	9.2	29.2	1.7	0.8
**AE subtype**	43.3	18.3	25	10	15
**The average**	14.48	20.94	32.16	5.46	15.24

Our observations revealed that X4 tropism varied among the studied subtypes; the most (43.3 %) and the least (5.8 %) X4 subtypes were related to the AE and C subtypes, respectively (Table 3).

**Table 3:** X4 tropism prediction rate.

Subtype and suggested predicting model	X4 (%)
CRF-35 AD (PhenoSeq)	5 (10.6 %)
A (PhenoSeq)	13 (10.8 %)
B (PSSM)	20 (16.7 %)
C (PSSM)	7 (5.8 %)
AE (PSSM)	52 (43.3 %)

Our analyses revealed that Geno2Pheno and Net charge presented the highest (82.1 %) and lowest (58.5 %) similarity results, respectively, among the various tools for all analyzed sequences (Table 4 and **Additional** Table 2 (a-e) for similarity results for each subtype and model.

**Table 4:**
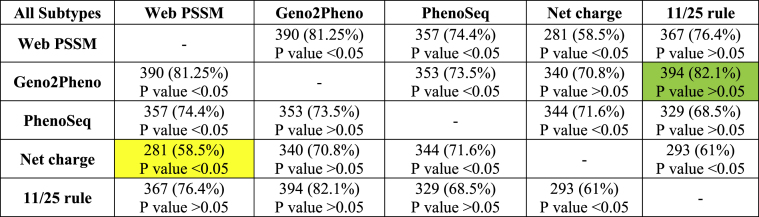
Most (green) and least (yellow) similar tropisms resulting from various tools.

**Fig. 5:**
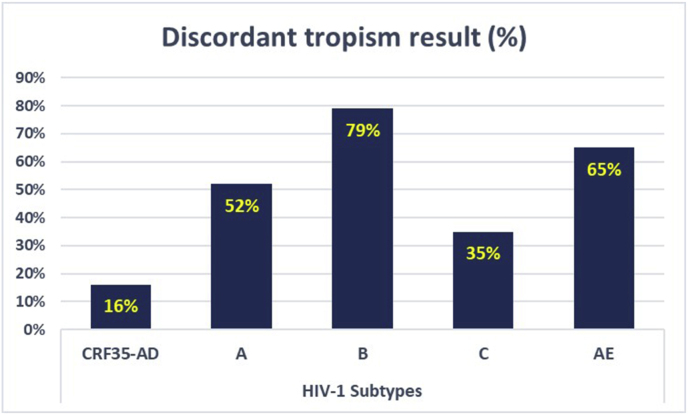
Discordant tropic results in the studied subtypes.

Among the investigated subtypes, a significant number of sequences presented conflicting results in quintuplicate tests. However, the level of diversity varied among the different genotypes. According to Fig. 5, the results varied significantly for different subtypes, with subtype B having the highest divergence at 79 %, followed by AE at 65 %, A at 52 %, C at 35 %, and CRF35-AD at 16 %.

**Fig. 6:**
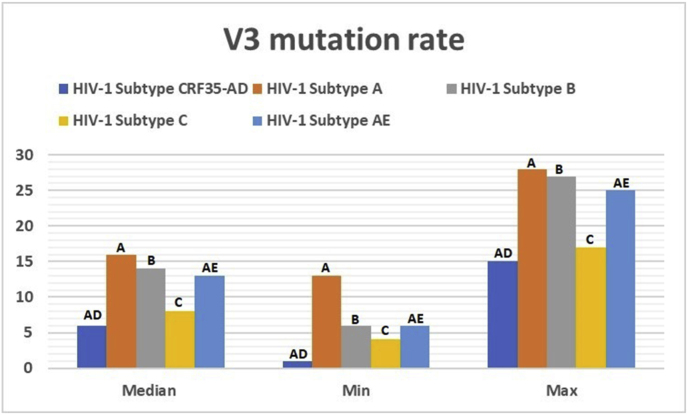
*V3* mutation rates in subtypes A, B, C, AE, and CRF35-AD.

The frequency of *V3* mutations in all sequences is summarized in Fig. 6. The findings revealed that subtype A had the median mutation rate at 16 (13–28), whereas CRF35-AD had the lowest mutation rate at 6 (1–15).

### 3.2 The patterns of crown motif, N-glycosylation, and T8V mutation in various subtypes and R5 and X4 viruses

**Fig. 7:**
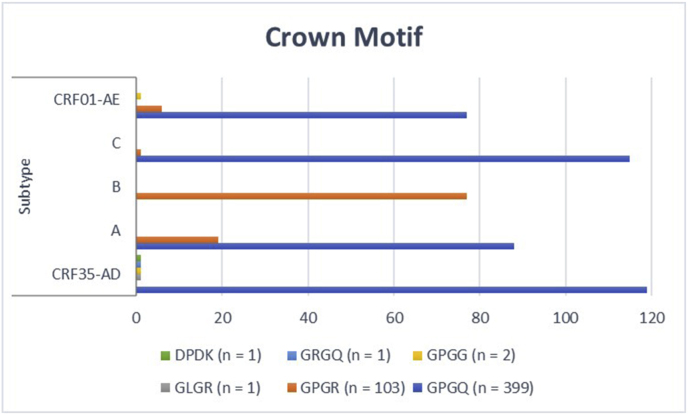
Variations in the crown motif of the *V3* region according to coreceptor tropism and subtype.

In addition to the typical crown motif (GPGQ), diverse crown motifs, including GPGG, GPGR, GRGQ, GLGR, and DPDK, were found in both the R5 and X4 strains of the studied sequences (Fig. 7). The prediction results of N-linked glycosylation, crown motif, and T8V of all the isolates are summarized in Table 5 and **Additional** Table 3 (a-e). Considering our data, R5 viruses in both subtypes A and B harbored slightly more crown motifs, whereas all five subtypes carried N-linked glycosylation positions.

**Table 5:** Prediction of N-glycosylation, T8V, and crown motif positions in the gp120 V3 loop.

	V3 Tropism	N-glycosylation	Crown motif^a^	T8V Mutation
Iranian samples **(CRF35-AD)**	R5:110 (89.4 %)	R5:101/103 (98.1 %)	R5:103/103 (100 %)	R5:0/103 (0 %)
	X4:13 (10.6 %)	X4:19/20 (95 %)	X4:20/20 (100 %)	X4:0/20 (0 %)
Subtype A (**Pheno Seq)**	R5:107 (89.2 %)	R5:106/107 (99.1 %)	R5:99/107 (92.5 %)	R5:0/107 (0 %)
	X4:13 (10.8 %)	X4:10/13 (76.9 %)	X4:8/13 (61.5 %)	X4:0/13 (0 %)
Subtype B (**Pheno Seq)**	R5: 53 (44.2 %)	R5:53/53 (100 %)	R5:53/53 (100 %)	R5:0/53 (0 %)
	X4: 67 (55.8 %)	X4:63/67 (94.1 %)	X4: 24/67 (35.8 %)	X4:0/67 (0 %)
Subtype C (**Web PSSM)**	R5: 113 (94.2 %)	R5: 111/113 (98.3 %)	R5: 110/113 (97.3 %)	R5: 0/113 (0 %)
	X4: 7 (5.8 %)	X4: 6/7 (85.7 %)	X4: 6/7 (85.7 %)	X4: 0/7 (0 %)
Subtype AE (**Web PSSM)**	R5: 68 (56.7 %)	R5: 66/68 (97.05 %)	R5: 50/68 (73.5 %)	R5: 0/68 (0 %)
	X4: 52 (43.3 %)	X4: 43/52 (82.7 %)	X4: 34/52 (65.4 %)	X4: 0/52 (0 %)

a Crown motif: GPGQ, GPGG, GPGR, GRGQ, GLGR, and DPDK.

## 4 Discussion

Bioinformatics tools are essential for monitoring disease outbreaks and investigating antimicrobial resistance. They assist in developing personalized medicine aimed at specific pathogens and hosts [46]. Additionally, these tools aid in identifying new disease antigens, improving diagnostic precision and treatment approaches [47]. CCR5 entry inhibitors may be ineffective and potentially harmful for patients with CXCR4-using *HIV-1* [30]. The development of an accurate and cost-effective tropism assay is crucial for determining *HIV-1* tropism and predicting the treatment response to CCR5 antagonists. X4 or R5X4 viruses often emerge in the later stages of *HIV-1* infection, particularly in some subtypes, such as subtype A, whereas R5 viruses are more common in the early stages of infection [43,48]. Here, the tropisms of the four major subtypes, A, B, C, and AE, and the CRF35-AD subtype were evaluated via five genotypic procedures. Since the analysis of three of the five bioinformatic tools (PhenoSeq, WebPSSM, and Geno2Pheno) is based on the sequence of the *HIV-1* B subtype [49], two more bioinformatic methods (Net charge and 11/25 rules) were used to define the *HIV-1* tropism of non-B subtypes of A, C, and AE to overcome the unsatisfactory sensitivity of each bioinformatic tool. As outlined previously for *V3* tropism prediction in Iranian patients with CRF35-AD [28], most patients had R5-tropic viral variants, which contrasts with previous findings for HIV-1-infected individuals [30,50] and subtype C infections, where X4 variants are less common than R5 variants [43,51]. The results suggest that the tropism prediction method may vary depending on the specific HIV subtype being studied. Subtype A was the first subtype that was assessed and is predominant in parts of East Africa, Russia, and other former Soviet Union countries [52]. Previous studies have shown that the majority of subtype A sequences are CCR5-tropic (R5), ranging from 60.8 % (73/120) to 100 % (120/120). It seems that the majority of retrieved sequences are R5 tropic, mostly due to the predominance of R5-tropic viral variants in *HIV-1* subtype A [16,45,53,54]. Two algorithms, Geno2Pheno (100 % R5) and WebPSSM (95 % R5), appear to provide more accurate predictions of viral tropism for subtype A [55] than other methods, such as Net charge, the 11/25 rule, and PhenoSeq [54].

The second subtype was B, which is the most widespread viral strain in North America, Australia, and Europe [52]. Our data suggest that the Geno2Pheno (92.5 % R5) model can predict more accurate tropism results for subtype B, as several studies reported that 70%–95 % of *HIV-1-*positive individuals are infected by R5-tropic *HIV-1* strains [56,57]. Importantly, the evaluation via Net charge and PhenoSeq software revealed a significant number of sequences exhibiting X4 tropism, suggesting that these two programs may not be the most effective tools for predicting tropism in subtype B and should be supplemented with other methods [54,58]. Furthermore, it has been reported that high percentages of the latent reservoir in subtype B use CCR5 [59]; supposedly, the treatment response to MVC for subtype B can be promising. Therefore, tropism screening of patients infected with the *HIV-1* B subtype is recommended to exclude patients with X4 tropism from CCR5 antagonist therapy.

Subtype C is the third predominant subtype, accounting for more than 50 % of all *HIV-1* infections worldwide, and is dominant in southern Africa [60] and India [52]. Some reports have shown that the percentage of X4 virus in subtype C is low (29.41 %) in patients with advanced AIDS [51,61]. Nevertheless, it is controversial whether infection with the C subtype causes faster progression to AIDS than other subtypes have been studied in large-scale reports [62,63]. Our results revealed that, with the exception of the net charge, the other methods used presented a low percentage of X4 tropism in subtype C, which is consistent with the findings of several other studies [61,64]. The WebPSSM and the 11/25 rule may be the best algorithms for tropism prediction in the C subtype, as applied in other studies [34,65]. Alternatively, the net charge is not recommended as a predictor of tropism in subtype C. Furthermore, if less than 5–10 % of *HIV-1* subtype C patients harbor X4 variants, routine tropism assessment may not be necessary [66], and R5 antagonists could be promising treatment options for this population. The last subtype analyzed here was CRF01-AE recombinant, which is the major subtype responsible for the pandemic in Asia, with a prevalence of 84 %, accounting for 4.6 % of total *HIV-1* infections worldwide [67]. One of the critical characteristics of the CRF01-AE subtype is the high prevalence of X4 coreceptor binding viruses in this subtype [24,25], with a distribution of 20 % [68]--40–70 % [24,69]. Several reports have shown that the high prevalence of X4-tropism in CRF01-AE recombinants is potentially associated with faster HIV/AIDS progression to AIDS [24,25,67]. Supposedly, CCR5 antagonists may not play a major role in the therapy of patients in geographic regions where the *HIV-1* CRF01-AE subtype is predominant unless tropism testing is performed before the prescription of CCR5 antagonists. On the basis of different studies, Geno2Pheno is the most valuable algorithm for *V3* prediction tropism for *HIV-1* CRF01-AE [57,67,70], and the treatment response to MVC was correlated with the tropism predicted by Geno2Pheno [57,71]. However, our findings revealed that Geno2Pheno predicted only 12/120 (10 %) of the CRF01-AE sequences as having X4 tropism, whereas WebPSSM predicted 52/120 (43.3 %) of the sequences as having X4 tropism. The PhenoSeq and Net charge models had similar percentages of approximately 20 %. A previous study by Ramond et al. revealed that the Geno2Pheno algorithm had high sensitivity (91 %) but low specificity (54 %), whereas the combined 11/25 and net charge rule had high specificity (98 %) but low sensitivity (64 %) [72]. Owing to the high prevalence of CXCR4-tropic variants in the AE subtype and the genotypic tools used in this study, WebPSSM may be a better tool for coreceptor tropism prediction in the AE subtype. This method has demonstrated greater agreement with phenotypic tests than alternative genotypic prediction methods do [73]. Additionally, according to previous research, the WebPSSM has specificity and sensitivity percentages of 94 and 88, respectively [[42], [43], [44]].

Various studies have shown that the CRF35-AD subtypes of *HIV-1* are prevalent in Iran and Afghanistan [74,75]. CRF35-AD has been recognized as the most common subtype found in high-risk populations in Iran, especially among intravenous drug users (IDUs) [76]. The mutation rate of *V3* was comparable in both the X4 and R5 tropisms in CRF35-AD samples. These findings indicate that *V3* mutations may not play a significant role in regulating *HIV-1* tropism or the risk of developing AIDS. Unlike our research, Delgado and colleagues reported that the genetic diversity of *HIV-1* in the *gp120-V3* region could influence tropism outcomes [77]. More research is recommended to determine the factors that affect HIV-1 tropism. According to our findings, the PhenoSeq method appears to be reliable for predicting the tropism of *HIV-1* CRF35-AD, accurately identifying 10.6 % of X4-tropic viruses. These findings suggest that this method is suitable for clinical use to guide the cautious use of CCR5 antagonists.

The prediction of viral tropism results for the B and non-B subtypes differs among diverse genotypic tools. Although genotypic methods are based on the B subtype, our data revealed that such methods showed the most discordant tropism results for subtype B and then for AE, A, C, and CRF35-AD. The V3 tropism between the HIV-1 subtypes and CRF35-AD is due to genetic and structural factors affecting co-receptor usage. CRF35-AD has a more consistent genetic makeup, leading to fewer disparities in tropism assessments. In contrast, subtype B patients present more mutations outside the V3 region that significantly impact co-receptor tropism [78,79]. In general, the study revealed that CRF35-AD originated from southern Iran within 4 years, whereas other subtypes were evaluated in various countries over 10 years, suggesting lower immigration rates in Iran. Consequently, tropism testing for different subtypes is essential in regions with low migration rates. The analysis revealed that HIV-infected individuals in Iran had R5 tropism and responded favorably to CCR5 inhibitor drugs. As noted above, all genotypic tools were designed on the basis of the clinical and experimental data of the B subtype; however, these five models are appropriate for the prediction of coreceptor usage in diverse subtypes.

In summary, previous reports confirmed that different *HIV-1* subtypes may have specific coreceptor preferences [80,81]. This may pose a challenge when new classes of CCR5 antagonists are introduced for treatment initiation. We found a relatively high frequency of CCR5 tropism among the A, B, C, and CRF35-AD subtypes, except for the AE subtype. Therefore, a good prognosis and treatment outcome will be promising if patients infected with one of the A, B, C, or CRF35-AD subtypes are treated with MVC or vicriviroc.

Furthermore, the X4 tropism prediction rates were diverse among the procedures and subtypes used; Net-charge and Geno2Pheno predicted the most (39.8 %) and the least (4.8 %) X4 tropism in all 603 sequences, respectively. Therefore, net charge is recommended in clinical settings for conservative prescription of CCR5 antagonists.

This study examined the effects of N-glycosylation, crown motifs, and T8 mutation in the V3 sequence on HIV-1 tropism. The R5 viruses in subtypes A and B presented a slightly greater frequency of crown motifs, which was in line with the findings of some studies [29,36]. However, crown motifs rarely lead to a tropism switch toward X4 viruses; therefore, the crown motif rarely affects *HIV-1* pathogenesis. Our data revealed that the GPGQ crown motif was present in all the subtypes except for subtype B, which is in accordance with the findings of Guo et al. Moreover, crown motifs are conserved in the studied subtypes, which is why such motifs induce an antibody response [29]; therefore, the inclusion of such a motif in the vaccine construct may improve the neutralization response. The other factor assessed here was the effect of the N-glycosylation of the *V3* sequence on *HIV-1* tropism. Several studies have shown that differences in *HIV-1* coreceptor usage and N-linked glycosylation patterns among various subtypes are affected by *HIV-1* sequences [82]. Our study revealed differences in N-glycosylation patterns among HIV-1 subtypes, with higher rates in R5 strains of subtype A, whereas previous studies reported higher frequencies in R5 isolates of subtypes A, B, and C and in X4 strains of subtypes D and E [83], which almost disagreed with our research. However, some reports have demonstrated that the rate of N-glycosylation in X4 strains is greater than that in R5 strains [36,84].

Mutations in *V3*-loop sequences usually lead to *HIV-1* escape from immune responses [85], which might not reduce *HIV-1* replication [16]. *HIV-1* genetic variability in the V3 region of the B subtype, CRF01-AE recombinant [77], and other subtypes may have a strong impact on the determination of tropism via genotypic tools. Although AE subtypes [24,25,67] may be associated with faster progression to AIDS, the frequencies of X4-V3 and R5-V3 mutations across subtypes are relatively low and similar, suggesting that the V3 mutation rate alone has a negligible effect on progression to AIDS.

In this study, a mutation in the T8 amino acid was not detected in the V3 protein of the five subtypes. It seems that the T8 mutation failed to influence *HIV-1* tropism, which was in contrast with a study that showed that the T8V mutation was associated with a tropism switch toward the CXCR4-tropics [86].

The identification of tropism in each genotype via various software programs can yield varying outcomes, indicating that the five software programs (PhenoSeq, WebPSSM, Geno2Pheno, Net charge, and 11/25 rules) may not be universally effective in detecting tropism for the A, B, C, AE, and CRF35-AD subtypes. The following are possible suggestions for improving genotypic methods to make them applicable in clinical settings. As mentioned earlier, the evaluation of *HIV-1* tropism via bioinformatics tools is more accurate for subtype B patients [87,88]. Thus, such methods for determining HIV tropism need to incorporate additional subtypes and clinical parameters to improve accuracy. Additionally, genotypic methods have limited sensitivity in predicting X4 tropism [88,89], potentially leading to misclassification of X4-using variants as R5-using [90]. Accordingly, the periodic application of next-generation sequencing, which can detect minority X4 variants that are below the detection limit, is beneficial [88]. Further investigations in large populations are warranted to confirm the subtype-specific tropism for the use of CCR5 antagonists in HIV-1 treatment [91]. Various cohorts should be subjected to genotypic tropism assays to determine the effectiveness of CCR5 antagonist drugs in patients infected with R5 viruses and those infected with X4 viruses. Additionally, it appears that conducting phenotypic studies to determine tropism is still essential in assessing genotyping findings [88]. It is suggested that viruses from the major subtypes be analyzed via both phenotype and genotype to determine which genotyping method is more accurate on the basis of phenotypic limitations.

## 5 Conclusions

With the introduction of CCR5 antagonists, accurate, rapid, user-friendly, and cost-effective tropism assays with minimal processing have gained increasing importance in the diagnosis of R5, X4, or dual-tropic viruses to formulate treatment strategies for *HIV-1*-infected patients. Compared with phenotypic assays, genotypic methods define *HIV-1* tropism as an applicable procedure in all clinical settings, even in resource-setting countries; however, such methods need to be improved to increase the sensitivity of X4 virus detection. According to our data, CCR5 inhibitors are recommended for the majority of *HIV-1*-infected patients, as R5 viruses are highly prevalent in the A, B, and C subtypes, which are the predominant *HIV-1* subtypes in the world. However, CCR5 may not be predominant in Asia, in which AE subtypes are dominant. Furthermore, WebPSSM may be an appropriate algorithm for the determination of *HIV-1* tropism in A, B, C, AE, and CRF35-AD subtypes. Although the data of the present report revealed that R5 viruses in the A and B subtypes are more susceptible to the formation of various crown motifs, this conclusion cannot be reached unless the evolution of these particular strains follows. Considering that Iranian HIV-1 patients have a high response rate to treatment with CCR5 inhibitors, it is recommended that Iranian health policymakers consider the use of these drugs for treating Iranian patients, even without tropism testing. Hence, given the absence of phenotypic validation, the authors should strongly advocate for follow-up studies using phenotypic assays or large cohorts to confirm these findings.

## Summary points

• V3 tropism in major HIV-1 subtypes and CRFs was examined via five genotypic bioinformatics tools: WebPSSM, Geno2Pheno, PhenoSeq, Net Charge, and the 11/25 rule.
• The high frequency of CCR5-tropism in Iranian HIV-1-infected patients revealed that the use of maraviroc may lead to a high rate of response to therapy.
• R5 viruses were highly prevalent among the A, B, C, and CRF35-AD subtypes, whereas X4 variants were commonly found in the AE subtype.
• The WebPSSM method may be suitable for identifying coreceptors in HIV-1 subtypes.

## Ethics approval and consent to participate

The research study obtained ethical approval from the ethics committee at Shiraz University of Medical Sciences.

## Consent for publication

Not applicable.

## Disclosure statement

The authors declare that they have no conflicts of interest.

## Author contributions

Conceptualization, AH., NKH., ZM. ; methodology, NKH., SHA., FS. ; software, NKH., AH., SHA. ; validation, ZM., NKH., SHA. ; formal analysis, NKH., SHA. ; Investigation; FS., ZM., NKH. ; resources, AH. ; data curation, FS. ; writing—original draft preparation, AH., NKH., SHA. ; writing—review and editing; AH., NKH. ; visualization, AH. ; supervision, AH., NKH. ; project administration, AH. ; funding acquisition, AH.

## Funding

The proposal code was 29300 that was verified by Shiraz University of Medical Sciences, Shiraz, Iran (IR.SUMS.MED.REC.1403.171). In this context, we confirm that we did not receive any funding from any organization for this research. All the activities were self-funded, and no funding was sought from other sources outside the institutions.

## Declaration of competing interest

The authors declare that they have no known competing financial interests or personal relationships that could have appeared to influence the work reported in this paper.
