# Supplementary material for: Prediction of the Co-receptor usage of the main worldwide HIV-1 subtypes, CRF, and CRF35-AD in Iranian patients via the five genotypic tools
Source: Biochem Biophys Rep. 2025 Feb 17;41:101939. doi: 10.1016/j.bbrep.2025.101939 (PMC11875805; doi:10.1016/j.bbrep.2025.101939)
Supplement: Multimedia component 1 [file mmc1.docx]

**Supplemental Table 1a:** Frequency of V3 tropism in subtype A.

| **#** | **AC number** | **Web PSSM** | **PhenoSeq** | **Net charge** | **Geno2Pheno** | **11/25 rule** | **Final Result** | **#** | **AC number** | **Web PSSM** | **PhenoSeq** | **Net charge** | **Geno2Pheno** | **11/25 rule** | **Final Result** |
| --- | --- | --- | --- | --- | --- | --- | --- | --- | --- | --- | --- | --- | --- | --- | --- |
| **1** | KC200418 | R5 | R5 | R5 | R5 | R5 | R5 | **61** | JF960069 | R5 | R5 | X4 | R5 | X4 | X4 |
| **2** | DQ396400 | R5 | R5 | R5 | R5 | R5 | R5 | **62** | JF959544 | R5 | R5 | R5 | R5 | R5 | R5 |
| **3** | JF958607 | R5 | R5 | R5 | R5 | R5 | R5 | **63** | JF959543 | R5 | R5 | R5 | R5 | R5 | R5 |
| **4** | JF958938 | R5 | R5 | R5 | R5 | R5 | R5 | **64** | JF960068 | R5 | X4 | X4 | R5 | X4 | X4 |
| **5** | KY825320 | R5 | R5 | R5 | R5 | X4 | X4 | **65** | HM246200 | R5 | R5 | X4 | R5 | R5 | X4 |
| **6** | KY825321 | R5 | R5 | X4 | R5 | X4 | X4 | **66** | HM239540 | R5 | R5 | R5 | R5 | R5 | R5 |
| **7** | KY825322 | R5 | R5 | R5 | R5 | R5 | R5 | **67** | JF960067 | R5 | R5 | X4 | R5 | X4 | X4 |
| **8** | JF958937 | R5 | R5 | R5 | R5 | R5 | R5 | **68** | GQ409975 | R5 | X4 | X4 | R5 | R5 | X4 |
| **9** | JF958936 | R5 | R5 | R5 | R5 | R5 | R5 | **69** | JF960066 | R5 | R5 | X4 | R5 | X4 | X4 |
| **10** | JF958935 | R5 | R5 | R5 | R5 | R5 | R5 | **70** | JF960065 | R5 | R5 | X4 | R5 | X4 | X4 |
| **11** | KY825323 | R5 | R5 | R5 | R5 | X4 | X4 | **71** | JF960064 | R5 | R5 | X4 | R5 | X4 | X4 |
| **12** | JF958934 | R5 | R5 | R5 | R5 | R5 | R5 | **72** | FJ832077 | R5 | R5 | X4 | R5 | R5 | X4 |
| **13** | KY825324 | R5 | R5 | X4 | R5 | X4 | X4 | **73** | JF960063 | R5 | R5 | X4 | R5 | X4 | X4 |
| **14** | KY825304 | R5 | R5 | R5 | R5 | R5 | R5 | **74** | JF960062 | R5 | R5 | X4 | R5 | X4 | X4 |
| **15** | KY825305 | R5 | R5 | R5 | R5 | R5 | R5 | **75** | JF960061 | R5 | R5 | X4 | R5 | X4 | X4 |
| **16** | JF958933 | R5 | R5 | R5 | R5 | R5 | R5 | **76** | FJ659314 | R5 | R5 | X4 | R5 | R5 | X4 |
| **17** | KY825306 | R5 | R5 | R5 | R5 | R5 | R5 | **77** | HM176760 | X4 | X4 | X4 | R5 | R5 | X4 |
| **18** | JF958932 | R5 | R5 | R5 | R5 | R5 | R5 | **78** | HM176761 | R5 | R5 | X4 | R5 | R5 | X4 |
| **19** | HM239624 | R5 | R5 | X4 | R5 | R5 | X4 | **79** | JF960060 | R5 | R5 | X4 | R5 | X4 | X4 |
| **20** | GQ401740 | R5 | R5 | X4 | R5 | R5 | X4 | **80** | JF960059 | R5 | R5 | X4 | X4 | R5 | X4 |
| **21** | GQ401724 | R5 | R5 | R5 | R5 | R5 | R5 | **81** | HQ385473 | R5 | R5 | R5 | R5 | R5 | R5 |
| **22** | GQ409981 | R5 | R5 | R5 | R5 | R5 | R5 | **82** | HM176759 | R5 | X4 | X4 | R5 | R5 | X4 |
| **23** | KY825307 | R5 | R5 | R5 | R5 | X4 | X4 | **83** | GU076168 | R5 | R5 | X4 | R5 | R5 | X4 |
| **24** | GQ401733 | R5 | R5 | X4 | R5 | R5 | X4 | **84** | HM246206 | X4 | R5 | X4 | R5 | R5 | X4 |
| **25** | GQ401731 | R5 | R5 | X4 | R5 | R5 | X4 | **85** | HM239551 | X4 | R5 | X4 | R5 | R5 | X4 |
| **26** | KY825308 | R5 | R5 | R5 | R5 | R5 | R5 | **86** | MF591927 | R5 | R5 | R5 | R5 | R5 | R5 |
| **27** | KY825309 | R5 | R5 | R5 | R5 | R5 | R5 | **87** | KR709173 | R5 | R5 | R5 | R5 | R5 | R5 |
| **28** | KY825310 | R5 | X4 | R5 | R5 | R5 | X4 | **88** | KY825325 | R5 | R5 | X4 | R5 | X4 | X4 |
| **29** | GQ401728 | R5 | R5 | R5 | R5 | R5 | R5 | **89** | KY825326 | R5 | R5 | R5 | R5 | R5 | R5 |
| **30** | FM955836 | R5 | R5 | R5 | R5 | R5 | R5 | **90** | KP696966 | X4 | R5 | X4 | R5 | R5 | X4 |
| **31** | FM955805 | R5 | R5 | X4 | R5 | R5 | X4 | **91** | KF247204 | R5 | R5 | R5 | R5 | R5 | R5 |
| **32** | EF143463 | R5 | X4 | R5 | R5 | X4 | X4 | **92** | KY825327 | R5 | R5 | R5 | R5 | X4 | X4 |
| **33** | EF143462 | R5 | R5 | R5 | R5 | R5 | R5 | **93** | KF429719 | R5 | R5 | X4 | R5 | R5 | X4 |
| **34** | JF959558 | R5 | R5 | R5 | R5 | R5 | R5 | **94** | KF429718 | R5 | R5 | X4 | R5 | R5 | X4 |
| **35** | JF959557 | R5 | R5 | R5 | R5 | R5 | R5 | **95** | KF429717 | R5 | R5 | R5 | R5 | R5 | R5 |
| **36** | JF959556 | R5 | R5 | R5 | R5 | R5 | R5 | **96** | KF429716 | R5 | R5 | X4 | R5 | R5 | X4 |
| **37** | KY825297 | R5 | R5 | R5 | R5 | X4 | X4 | **97** | KY825298 | R5 | R5 | R5 | R5 | X4 | X4 |
| **38** | JF959555 | R5 | R5 | R5 | R5 | R5 | R5 | **98** | KY825317 | R5 | R5 | R5 | R5 | R5 | R5 |
| **39** | KY825311 | R5 | R5 | R5 | R5 | R5 | R5 | **99** | KY825299 | R5 | R5 | X4 | R5 | X4 | X4 |
| **40** | KY825312 | R5 | R5 | X4 | R5 | X4 | X4 | **100** | KY825328 | R5 | R5 | X4 | R5 | X4 | X4 |
| **41** | JF959554 | R5 | X4 | R5 | R5 | R5 | X4 | **101** | KY825300 | R5 | R5 | R5 | R5 | R5 | R5 |
| **42** | JF959553 | R5 | R5 | X4 | X4 | R5 | X4 | **102** | KY825301 | R5 | R5 | R5 | R5 | R5 | R5 |
| **43** | KY825313 | R5 | R5 | R5 | R5 | X4 | X4 | **103** | KY825329 | R5 | R5 | R5 | R5 | R5 | R5 |
| **44** | JF959552 | R5 | R5 | R5 | R5 | R5 | R5 | **104** | KY825302 | R5 | R5 | R5 | R5 | R5 | R5 |
| **45** | JF959551 | R5 | R5 | R5 | R5 | R5 | R5 | **105** | KY825330 | R5 | R5 | X4 | R5 | X4 | X4 |
| **46** | KY825314 | R5 | X4 | R5 | R5 | R5 | X4 | **106** | KY825303 | R5 | X4 | R5 | R5 | R5 | X4 |
| **47** | KY825315 | R5 | R5 | R5 | R5 | R5 | R5 | **107** | KY825318 | R5 | R5 | R5 | R5 | R5 | R5 |
| **48** | JF959550 | R5 | R5 | R5 | R5 | R5 | R5 | **108** | KY825331 | R5 | R5 | R5 | R5 | X4 | X4 |
| **49** | JF959549 | R5 | R5 | R5 | R5 | R5 | R5 | **109** | FJ832080 | R5 | X4 | X4 | R5 | R5 | X4 |
| **50** | KY825316 | R5 | R5 | X4 | R5 | X4 | X4 | **110** | MF581058 | R5 | R5 | R5 | R5 | X4 | X4 |
| **51** | MH627127 | X4 | X4 | X4 | R5 | R5 | X4 | **111** | KY825332 | R5 | R5 | R5 | R5 | X4 | X4 |
| **52** | .JF959548 | R5 | R5 | R5 | R5 | R5 | R5 | **112** | MF941096 | R5 | R5 | R5 | R5 | R5 | R5 |
| **53** | JF959547 | R5 | R5 | R5 | R5 | R5 | R5 | **113** | KU685559 | R5 | X4 | R5 | R5 | R5 | X4 |
| **54** | KC200478 | X4 | X4 | R5 | R5 | R5 | X4 | **114** | KU685558 | R5 | X4 | X4 | R5 | R5 | X4 |
| **55** | JF959546 | R5 | R5 | R5 | R5 | R5 | R5 | **115** | KY825333 | R5 | R5 | R5 | R5 | R5 | R5 |
| **56** | JF960073 | R5 | R5 | X4 | R5 | X4 | X4 | **116** | KY825334 | R5 | R5 | X4 | R5 | X4 | X4 |
| **57** | JF960072 | R5 | R5 | X4 | R5 | X4 | X4 | **117** | KY825335 | R5 | R5 | R5 | R5 | R5 | R5 |
| **58** | JF960071 | R5 | R5 | X4 | R5 | X4 | X4 | **118** | MK732771 | R5 | R5 | R5 | R5 | R5 | R5 |
| **59** | JF959545 | R5 | R5 | R5 | R5 | R5 | R5 | **119** | KY825319 | R5 | R5 | X4 | R5 | X4 | X4 |
| **60** | JF960070 | R5 | R5 | X4 | R5 | X4 | X4 | **120** | OM240733 | R5 | R5 | R5 | R5 | R5 | R5 |
|  | | **WebPSSM** | | **PhenoSeq** | | **Net charge** | | **Geno2Pheno** | | **11/25 rule** | | **Final Result** | | | |
| **Frequency** | | R5:114 (95%)  X4:6 (5%) | | R5:107 (89.2%)  X4:13 (10.8%) | | R5: 73 (60.8%)  X4: 43 (39.2%) | | R5: 120 (100%) | | R5: 83 (69.2%)  X4:37 (30.8%) | | R5: 57 (47.5%)  X4: 63 (52.5%) | | | |

**Supplemental Table 1b:** Frequency of V3 tropism in subtype B

| **#** | **AC number** | **Web PSSM** | **PhenoSeq** | **Net charge** | **Geno2Pheno** | **11/25 rule** | **Final Result** | **#** | **AC number** | **Web PSSM** | **PhenoSeq** | **Net charge** | **Geno2Pheno** | **11/25 rule** | **Final Result** |
| --- | --- | --- | --- | --- | --- | --- | --- | --- | --- | --- | --- | --- | --- | --- | --- |
| **1** | MW865570 | R5 | R5 | R5 | R5 | R5 | R5 | **61** | MW881640 | X4 | X4 | X4 | R5 | R5 | X4 |
| **2** | MW660559 | R5 | R5 | R5 | R5 | R5 | R5 | **62** | OK075245 | R5 | X4 | X4 | R5 | R5 | X4 |
| **3** | MW660558 | R5 | R5 | R5 | R5 | R5 | R5 | **63** | OK075240 | R5 | X4 | X4 | R5 | R5 | X4 |
| **4** | MW660557 | R5 | R5 | R5 | R5 | R5 | R5 | **64** | MW881636 | R5 | X4 | R5 | R5 | R5 | X4 |
| **5** | OK514759 | R5 | X4 | R5 | R5 | R5 | X4 | **65** | OK075239 | R5 | R5 | X4 | R5 | R5 | X4 |
| **6** | OK075266 | X4 | X4 | X4 | R5 | X4 | X4 | **66** | OK075236 | R5 | X4 | R5 | R5 | R5 | X4 |
| **7** | MW881626 | R5 | R5 | X4 | R5 | R5 | X4 | **67** | MW881635 | R5 | X4 | X4 | R5 | X4 | X4 |
| **8** | MW660556 | R5 | R5 | R5 | R5 | R5 | R5 | **68** | MW881645 | X4 | X4 | X4 | R5 | R5 | X4 |
| **9** | MW865554 | R5 | R5 | X4 | R5 | R5 | X4 | **69** | MW881634 | R5 | X4 | X4 | R5 | X4 | X4 |
| **10** | MW865542 | R5 | R5 | X4 | R5 | R5 | X4 | **70** | MW881633 | R5 | X4 | R5 | R5 | R5 | X4 |
| **11** | MW881625 | R5 | X4 | X4 | X4 | X4 | X4 | **71** | MW881622 | R5 | X4 | X4 | R5 | R5 | X4 |
| **12** | MW865541 | R5 | R5 | X4 | R5 | R5 | X4 | **72** | MW881644 | R5 | X4 | X4 | R5 | R5 | X4 |
| **13** | MW881624 | R5 | X4 | R5 | R5 | R5 | X4 | **73** | MW881621 | X4 | X4 | X4 | R5 | R5 | X4 |
| **14** | MW881623 | R5 | X4 | R5 | R5 | R5 | X4 | **74** | MW881620 | X4 | X4 | X4 | R5 | R5 | X4 |
| **15** | OL446078 | R5 | X4 | R5 | R5 | R5 | X4 | **75** | MW881619 | X4 | X4 | X4 | R5 | R5 | X4 |
| **16** | MW865540 | R5 | R5 | X4 | R5 | R5 | X4 | **76** | MW881642 | R5 | X4 | X4 | R5 | R5 | X4 |
| **17** | OK514777 | X4 | X4 | X4 | R5 | R5 | X4 | **77** | MW865577 | R5 | R5 | X4 | R5 | R5 | X4 |
| **18** | MW865539 | R5 | R5 | X4 | R5 | R5 | X4 | **78** | MW865576 | R5 | R5 | X4 | R5 | R5 | X4 |
| **19** | MW865538 | R5 | R5 | X4 | R5 | R5 | X4 | **79** | OK514757 | R5 | X4 | X4 | X4 | R5 | X4 |
| **20** | MW865537 | R5 | X4 | X4 | R5 | R5 | X4 | **80** | OK514756 | R5 | X4 | X4 | R5 | R5 | X4 |
| **21** | MW865536 | R5 | X4 | X4 | R5 | R5 | X4 | **81** | MW881738 | R5 | R5 | X4 | R5 | R5 | X4 |
| **22** | MW865535 | R5 | X4 | X4 | R5 | R5 | X4 | **82** | MW865575 | R5 | R5 | X4 | R5 | R5 | X4 |
| **23** | OK514776 | X4 | X4 | X4 | R5 | R5 | X4 | **83** | MW865574 | R5 | R5 | X4 | R5 | R5 | X4 |
| **24** | MW865534 | R5 | X4 | X4 | R5 | R5 | X4 | **84** | MW865573 | R5 | R5 | X4 | R5 | R5 | X4 |
| **25** | MW660576 | R5 | R5 | R5 | R5 | R5 | R5 | **85** | MW865572 | R5 | R5 | X4 | R5 | R5 | X4 |
| **26** | OK514775 | X4 | X4 | X4 | R5 | R5 | X4 | **86** | MW881733 | R5 | X4 | X4 | R5 | X4 | X4 |
| **27** | OK514774 | X4 | X4 | X4 | R5 | R5 | X4 | **87** | MW881632 | X4 | X4 | X4 | R5 | X4 | X4 |
| **28** | OK514761 | R5 | X4 | X4 | R5 | R5 | X4 | **88** | MW865571 | R5 | R5 | X4 | R5 | R5 | X4 |
| **29** | MW660575 | R5 | R5 | R5 | R5 | R5 | R5 | **89** | MW865532 | R5 | X4 | X4 | R5 | R5 | X4 |
| **30** | MW660574 | R5 | R5 | R5 | R5 | R5 | R5 | **90** | MW881631 | X4 | X4 | X4 | R5 | X4 | X4 |
| **31** | MW660573 | R5 | R5 | R5 | R5 | R5 | R5 | **91** | MW881618 | R5 | X4 | X4 | R5 | R5 | X4 |
| **32** | MW660572 | R5 | R5 | R5 | R5 | R5 | R5 | **92** | MW865531 | R5 | X4 | X4 | R5 | R5 | X4 |
| **33** | MW660571 | R5 | R5 | R5 | R5 | R5 | R5 | **93** | MW660581 | R5 | R5 | R5 | X4 | R5 | X4 |
| **34** | MW660570 | R5 | R5 | R5 | R5 | R5 | R5 | **94** | MW660582 | R5 | R5 | R5 | X4 | R5 | X4 |
| **35** | MW660569 | R5 | R5 | R5 | R5 | R5 | R5 | **95** | MW660545 | R5 | R5 | R5 | R5 | R5 | R5 |
| **36** | MW660568 | R5 | R5 | R5 | R5 | R5 | R5 | **96** | MW660544 | R5 | R5 | R5 | R5 | R5 | R5 |
| **37** | OK514758 | X4 | X4 | X4 | R5 | X4 | X4 | **97** | MW660543 | R5 | R5 | R5 | X4 | R5 | X4 |
| **38** | MW660567 | R5 | R5 | R5 | R5 | R5 | R5 | **98** | MW405288 | R5 | R5 | R5 | R5 | R5 | R5 |
| **39** | OK514755 | R5 | X4 | X4 | R5 | R5 | X4 | **99** | MW262784 | R5 | R5 | X4 | R5 | R5 | X4 |
| **40** | OK514754 | R5 | X4 | X4 | R5 | R5 | X4 | **100** | MW865530 | R5 | X4 | X4 | R5 | R5 | X4 |
| **41** | MW660566 | R5 | R5 | R5 | R5 | R5 | R5 | **101** | MW262783 | R5 | X4 | R5 | X4 | X4 | X4 |
| **42** | MW660555 | R5 | R5 | R5 | R5 | R5 | R5 | **102** | MW262782 | R5 | X4 | R5 | X4 | X4 | X4 |
| **43** | OK075282 | X4 | X4 | X4 | R5 | X4 | X4 | **103** | MW865529 | R5 | X4 | X4 | R5 | R5 | X4 |
| **44** | MW660548 | R5 | R5 | R5 | R5 | R5 | R5 | **104** | MW262781 | R5 | R5 | X4 | R5 | R5 | X4 |
| **45** | OK075259 | R5 | X4 | R5 | R5 | R5 | X4 | **105** | MW865528 | R5 | X4 | X4 | R5 | R5 | X4 |
| **46** | OK075275 | R5 | X4 | X4 | R5 | R5 | X4 | **106** | OK514771 | R5 | R5 | X4 | R5 | R5 | X4 |
| **47** | OK075274 | R5 | X4 | X4 | R5 | R5 | X4 | **107** | MW865527 | R5 | X4 | X4 | X4 | R5 | X4 |
| **48** | OK075257 | X4 | X4 | X4 | R5 | R5 | X4 | **108** | MW865526 | R5 | X4 | X4 | R5 | R5 | X4 |
| **49** | OK075255 | X4 | X4 | X4 | R5 | R5 | X4 | **109** | MZ427713 | R5 | X4 | X4 | R5 | R5 | X4 |
| **50** | OK075273 | R5 | X4 | X4 | R5 | R5 | X4 | **110** | MZ427708 | R5 | X4 | R5 | R5 | R5 | X4 |
| **51** | OK075267 | R5 | X4 | X4 | R5 | X4 | X4 | **111** | MW865525 | R5 | R5 | X4 | R5 | R5 | X4 |
| **52** | OK075253 | R5 | R5 | R5 | R5 | X4 | X4 | **112** | MW881735 | R5 | R5 | X4 | R5 | R5 | X4 |
| **53** | OK075252 | R5 | R5 | R5 | R5 | R5 | R5 | **113** | MW881676 | R5 | R5 | X4 | R5 | R5 | X4 |
| **54** | OK075265 | R5 | X4 | X4 | R5 | X4 | X4 | **114** | MW881670 | R5 | X4 | X4 | R5 | R5 | X4 |
| **55** | OK075247 | R5 | X4 | R5 | R5 | R5 | X4 | **115** | MW660547 | R5 | R5 | R5 | R5 | R5 | R5 |
| **56** | OK075263 | X4 | X4 | X4 | R5 | X4 | X4 | **116** | MW660546 | R5 | R5 | R5 | R5 | R5 | R5 |
| **57** | OK075262 | R5 | R5 | X4 | R5 | R5 | X4 | **117** | OK514772 | R5 | R5 | X4 | R5 | R5 | X4 |
| **58** | OK075260 | R5 | R5 | R5 | X4 | R5 | X4 | **118** | MT559049 | X4 | X4 | X4 | R5 | R5 | X4 |
| **59** | OK075246 | R5 | X4 | X4 | R5 | R5 | X4 | **119** | OK514763 | R5 | X4 | X4 | R5 | R5 | X4 |
| **60** | MW881641 | X4 | X4 | X4 | R5 | R5 | X4 | **120** | MT559048 | X4 | X4 | X4 | R5 | R5 | X4 |
|  | | **WebPSSM** | | **PhenoSeq** | | **Net charge** | | **Geno2Pheno** | | **11/25 rule** | | | **Final Result** | | |
| **Frequency** | | R5:100 (83.3%)  X4: 20 (16.7%) | | R5:53 (44.2%)  X4:67 (55.8%) | | R5: 41 (34.2%)  X4: 79 (65.8%) | | R5: 111 (92.5%)  X4: 9 (7.5%) | | R5:104 (86.7%)  X4:16 (13.3%) | | | R5: 25 (20.8%)  X4: 95 (79.2%) | | |

**Supplemental Table 1c:** Frequency of V3 tropism in subtype C.

| **#** | **AC number** | **Web PSSM** | **PhenoSeq** | **Net charge** | **Geno2Pheno** | **11/25 rule** | **Final Result** | **#** | **AC number** | **Web PSSM** | **PhenoSeq** | **Net charge** | **Geno2Pheno** | **11/25 rule** | **Final Result** |
| --- | --- | --- | --- | --- | --- | --- | --- | --- | --- | --- | --- | --- | --- | --- | --- |
| **1** | MK561568 | R5 | R5 | R5 | R5 | R5 | R5 | **61** | MN703381 | R5 | R5 | R5 | R5 | R5 | R5 |
| **2** | MK041553 | R5 | R5 | R5 | R5 | R5 | R5 | **62** | MN275973 | R5 | R5 | R5 | R5 | R5 | R5 |
| **3** | MG836969 | R5 | X4 | R5 | R5 | R5 | X4 | **63** | MN275972 | R5 | R5 | R5 | R5 | R5 | R5 |
| **4** | MN387767 | R5 | R5 | R5 | R5 | R5 | R5 | **64** | MN387788 | R5 | R5 | X4 | R5 | R5 | X4 |
| **5** | MK561589 | R5 | R5 | R5 | R5 | R5 | R5 | **65** | MN275971 | R5 | R5 | R5 | R5 | R5 | R5 |
| **6** | MK561588 | R5 | R5 | R5 | R5 | R5 | R5 | **66** | MN275970 | R5 | R5 | R5 | R5 | R5 | R5 |
| **7** | MK561587 | R5 | R5 | R5 | R5 | R5 | R5 | **67** | MN387785 | R5 | R5 | R5 | R5 | R5 | R5 |
| **8** | MN387766 | R5 | R5 | R5 | R5 | R5 | R5 | **68** | MN275969 | R5 | R5 | X4 | R5 | R5 | X4 |
| **9** | MN387762 | R5 | X4 | R5 | R5 | R5 | X4 | **69** | MN387784 | R5 | R5 | X4 | R5 | R5 | X4 |
| **10** | MN703400 | X4 | X4 | X4 | R5 | R5 | X4 | **70** | MN387782 | R5 | R5 | X4 | R5 | R5 | X4 |
| **11** | MK561586 | R5 | R5 | R5 | R5 | R5 | R5 | **71** | MN387776 | R5 | R5 | R5 | X4 | R5 | X4 |
| **12** | MN703399 | X4 | R5 | X4 | R5 | R5 | X4 | **72** | MN275968 | R5 | R5 | R5 | R5 | R5 | R5 |
| **13** | MK458833 | R5 | R5 | R5 | R5 | R5 | R5 | **73** | MN387775 | R5 | R5 | R5 | R5 | R5 | R5 |
| **14** | MW197417 | R5 | X4 | R5 | R5 | R5 | X4 | **74** | MN387774 | R5 | X4 | X4 | R5 | R5 | X4 |
| **15** | MW197416 | R5 | R5 | R5 | R5 | R5 | R5 | **75** | MN387772 | R5 | R5 | R5 | R5 | R5 | R5 |
| **16** | MN703398 | X4 | R5 | X4 | R5 | R5 | X4 | **76** | MN275966 | R5 | R5 | R5 | R5 | R5 | R5 |
| **17** | MW197415 | R5 | R5 | R5 | R5 | R5 | R5 | **77** | MN275965 | R5 | R5 | R5 | R5 | R5 | R5 |
| **18** | MN703397 | X4 | R5 | X4 | R5 | R5 | X4 | **78** | MN275964 | R5 | R5 | X4 | R5 | R5 | X4 |
| **19** | MN703385 | R5 | R5 | X4 | R5 | R5 | X4 | **79** | MN387769 | R5 | R5 | R5 | R5 | R5 | R5 |
| **20** | MN703384 | R5 | R5 | R5 | R5 | R5 | R5 | **80** | MN387768 | R5 | R5 | R5 | R5 | R5 | R5 |
| **21** | MN387759 | R5 | R5 | R5 | R5 | R5 | R5 | **81** | MN387756 | R5 | R5 | R5 | R5 | R5 | R5 |
| **22** | MN387758 | R5 | X4 | R5 | R5 | R5 | X4 | **82** | MN275962 | R5 | R5 | R5 | R5 | R5 | R5 |
| **23** | MW197414 | R5 | R5 | R5 | R5 | R5 | R5 | **83** | MN275961 | R5 | R5 | R5 | R5 | R5 | R5 |
| **24** | MN387757 | R5 | R5 | R5 | R5 | R5 | R5 | **84** | MN275960 | R5 | R5 | X4 | R5 | R5 | X4 |
| **25** | MN387754 | R5 | R5 | X4 | R5 | R5 | X4 | **85** | MN275959 | R5 | R5 | X4 | R5 | R5 | X4 |
| **26** | MW197413 | R5 | R5 | X4 | R5 | R5 | X4 | **86** | MZ147137 | R5 | X4 | X4 | R5 | X4 | X4 |
| **27** | MW197412 | R5 | R5 | X4 | R5 | R5 | X4 | **87** | MZ147118 | R5 | R5 | X4 | R5 | R5 | X4 |
| **28** | MW197411 | X4 | R5 | R5 | R5 | R5 | X4 | **88** | MN275958 | R5 | R5 | X4 | R5 | R5 | X4 |
| **29** | MN387753 | R5 | R5 | R5 | R5 | R5 | R5 | **89** | MN275957 | R5 | R5 | R5 | R5 | R5 | R5 |
| **30** | MN387752 | R5 | R5 | R5 | R5 | R5 | R5 | **90** | MZ147116 | R5 | X4 | R5 | R5 | R5 | X4 |
| **31** | MN387750 | R5 | R5 | R5 | R5 | R5 | R5 | **91** | MN703396 | R5 | R5 | R5 | R5 | R5 | R5 |
| **32** | MN387748 | R5 | X4 | X4 | R5 | R5 | X4 | **92** | MN275956 | R5 | R5 | R5 | R5 | R5 | R5 |
| **33** | MN387747 | R5 | R5 | R5 | R5 | R5 | R5 | **93** | MN703395 | R5 | R5 | R5 | R5 | R5 | R5 |
| **34** | MN387746 | R5 | R5 | R5 | R5 | R5 | R5 | **94** | MN703394 | R5 | R5 | R5 | R5 | R5 | R5 |
| **35** | MN387745 | R5 | R5 | R5 | R5 | R5 | R5 | **95** | MN703393 | R5 | R5 | R5 | R5 | R5 | R5 |
| **36** | MN387744 | R5 | R5 | R5 | R5 | R5 | R5 | **96** | MN703392 | R5 | R5 | R5 | R5 | R5 | R5 |
| **37** | MW183667 | R5 | X4 | X4 | R5 | R5 | X4 | **97** | MN703391 | R5 | R5 | R5 | R5 | R5 | R5 |
| **38** | MN387740 | R5 | R5 | R5 | R5 | R5 | R5 | **98** | MN703366 | R5 | R5 | R5 | R5 | R5 | R5 |
| **39** | MW016028 | R5 | R5 | R5 | R5 | R5 | R5 | **99** | MN703365 | R5 | R5 | R5 | R5 | R5 | R5 |
| **40** | MW016027 | R5 | R5 | R5 | R5 | R5 | R5 | **100** | MK561599 | R5 | R5 | R5 | R5 | R5 | R5 |
| **41** | MN275984 | R5 | R5 | R5 | R5 | R5 | R5 | **101** | MN703364 | R5 | R5 | R5 | R5 | R5 | R5 |
| **42** | MN275983 | R5 | R5 | X4 | R5 | R5 | X4 | **102** | MN703363 | R5 | R5 | R5 | R5 | R5 | R5 |
| **43** | MW016026 | R5 | R5 | X4 | R5 | R5 | X4 | **103** | MK561598 | R5 | R5 | X4 | X4 | R5 | X4 |
| **44** | MN275982 | X4 | R5 | R5 | R5 | R5 | X4 | **104** | MN703362 | R5 | R5 | R5 | R5 | R5 | R5 |
| **45** | MN275980 | R5 | R5 | R5 | R5 | R5 | R5 | **105** | MK561597 | R5 | R5 | R5 | R5 | R5 | R5 |
| **46** | MW016025 | R5 | R5 | R5 | R5 | R5 | R5 | **106** | MN703361 | R5 | R5 | X4 | R5 | R5 | X4 |
| **47** | MW016024 | R5 | R5 | R5 | R5 | R5 | R5 | **107** | MK561596 | R5 | R5 | R5 | R5 | R5 | R5 |
| **48** | MN275979 | R5 | R5 | X4 | R5 | R5 | X4 | **108** | MK561595 | R5 | R5 | X4 | R5 | R5 | X4 |
| **49** | MN275978 | R5 | R5 | X4 | R5 | R5 | X4 | **109** | MN703360 | R5 | R5 | R5 | R5 | R5 | R5 |
| **50** | MW016023 | R5 | R5 | R5 | R5 | R5 | R5 | **110** | MZ642273 | R5 | R5 | X4 | R5 | R5 | X4 |
| **51** | MN703404 | R5 | R5 | X4 | R5 | R5 | X4 | **111** | MK561594 | R5 | R5 | X4 | R5 | R5 | X4 |
| **52** | MN275977 | R5 | R5 | X4 | R5 | R5 | X4 | **112** | MW262775 | R5 | R5 | R5 | R5 | R5 | R5 |
| **53** | MN275976 | R5 | R5 | R5 | R5 | R5 | R5 | **113** | MW262774 | R5 | R5 | R5 | R5 | R5 | R5 |
| **54** | MN703403 | R5 | R5 | X4 | R5 | R5 | X4 | **114** | MW262773 | R5 | R5 | R5 | R5 | R5 | R5 |
| **55** | MN275975 | R5 | R5 | R5 | R5 | R5 | R5 | **115** | MK561593 | R5 | R5 | R5 | R5 | R5 | R5 |
| **56** | MN703402 | R5 | R5 | X4 | R5 | R5 | X4 | **116** | MK561592 | R5 | R5 | R5 | R5 | R5 | R5 |
| **57** | MN703401 | X4 | R5 | X4 | R5 | R5 | X4 | **117** | MK561591 | R5 | R5 | R5 | R5 | R5 | R5 |
| **58** | MN703383 | R5 | R5 | R5 | R5 | R5 | R5 | **118** | MW262772 | R5 | R5 | R5 | R5 | R5 | R5 |
| **59** | MN275974 | R5 | X4 | X4 | R5 | R5 | X4 | **119** | MK561590 | R5 | R5 | R5 | R5 | R5 | R5 |
| **60** | MN703382 | R5 | R5 | R5 | R5 | R5 | R5 | **120** | MW262771 | R5 | R5 | R5 | R5 | R5 | R5 |
|  | | **WebPSSM** | | **PhenoSeq** | | **Net charge** | | **Geno2Pheno** | | **11/25 rule** | | | **Final Result** | | |
| **Frequency** | | R5:113 (94.2%)  X4:7 (5.8%) | | R5:109 (90.8%)  X4:11 (9.2%) | | R5: 85 (70.8%)  X4:35 (29.2%) | | R5: 118 (98.3%)  X4: 2 (1.7%) | | R5:119 (99.2%)  X4:1 (0.8%) | | | R5: 78 (65%)  X4: 42 (35%) | | |

**Supplemental Table 1d:** Frequency of V3 tropism in subtype AE

| **#** | **AC number** | **Web PSSM** | **PhenoSeq** | **Net charge** | **Geno2Pheno** | **11/25 rule** | **Final Result** | **#** | **AC number** | **Web PSSM** | **PhenoSeq** | **Net charge** | **Geno2Pheno** | **11/25 rule** | **Final Result** |
| --- | --- | --- | --- | --- | --- | --- | --- | --- | --- | --- | --- | --- | --- | --- | --- |
| **1** | MT163615 | R5 | R5 | X4 | R5 | R5 | X4 | **61** | MZ736182 | X4 | R5 | R5 | R5 | R5 | X4 |
| **2** | MT489625 | X4 | R5 | R5 | R5 | X4 | X4 | **62** | MZ736271 | R5 | R5 | R5 | R5 | R5 | R5 |
| **3** | MN387763 | R5 | R5 | X4 | R5 | R5 | X4 | **63** | MZ736270 | R5 | R5 | R5 | R5 | R5 | R5 |
| **4** | MT489589 | R5 | R5 | X4 | R5 | R5 | X4 | **64** | MZ736181 | X4 | R5 | R5 | R5 | R5 | X4 |
| **5** | MZ736248 | X4 | R5 | R5 | R5 | X4 | X4 | **65** | MZ736269 | R5 | R5 | R5 | R5 | R5 | R5 |
| **6** | MZ736247 | X4 | R5 | R5 | R5 | X4 | X4 | **66** | MZ736268 | R5 | R5 | R5 | R5 | R5 | R5 |
| **7** | MZ736246 | X4 | R5 | R5 | R5 | X4 | X4 | **67** | MZ736180 | X4 | R5 | R5 | R5 | R5 | X4 |
| **8** | MN387778 | R5 | R5 | X4 | X4 | R5 | X4 | **68** | MZ736267 | R5 | R5 | R5 | R5 | R5 | R5 |
| **9** | MT489620 | X4 | R5 | X4 | X4 | R5 | X4 | **69** | MZ736179 | X4 | R5 | R5 | R5 | R5 | X4 |
| **10** | MT489619 | X4 | R5 | X4 | X4 | R5 | X4 | **70** | MZ736175 | X4 | R5 | R5 | R5 | R5 | X4 |
| **11** | MZ736245 | X4 | R5 | R5 | X4 | X4 | X4 | **71** | MZ736174 | X4 | R5 | R5 | R5 | R5 | X4 |
| **12** | MT489616 | R5 | R5 | R5 | R5 | R5 | R5 | **72** | MZ736266 | R5 | R5 | R5 | R5 | R5 | R5 |
| **13** | MZ736244 | R5 | R5 | R5 | R5 | R5 | R5 | **73** | MZ736173 | X4 | R5 | R5 | R5 | R5 | X4 |
| **14** | MZ736243 | X4 | R5 | R5 | R5 | R5 | X4 | **74** | MZ736172 | X4 | R5 | R5 | R5 | R5 | X4 |
| **15** | MZ736242 | X4 | R5 | R5 | R5 | X4 | X4 | **75** | MZ736171 | X4 | R5 | R5 | R5 | R5 | X4 |
| **16** | MT489602 | R5 | R5 | R5 | R5 | R5 | R5 | **76** | MZ736265 | R5 | R5 | R5 | R5 | R5 | R5 |
| **17** | MZ736241 | X4 | R5 | R5 | R5 | X4 | X4 | **77** | MZ736264 | R5 | X4 | X4 | R5 | R5 | X4 |
| **18** | MT489595 | X4 | X4 | R5 | R5 | R5 | X4 | **78** | MZ736263 | R5 | X4 | X4 | R5 | R5 | X4 |
| **19** | MT489594 | R5 | X4 | R5 | R5 | R5 | X4 | **79** | MZ736159 | R5 | R5 | R5 | R5 | R5 | R5 |
| **20** | MT489593 | X4 | X4 | X4 | R5 | R5 | X4 | **80** | MZ736158 | R5 | R5 | R5 | R5 | R5 | R5 |
| **21** | MT489587 | X4 | X4 | X4 | X4 | R5 | X4 | **81** | MZ736157 | R5 | R5 | R5 | R5 | R5 | R5 |
| **22** | MT489584 | X4 | R5 | R5 | X4 | R5 | X4 | **82** | MZ736262 | R5 | X4 | X4 | R5 | R5 | X4 |
| **23** | MZ736240 | X4 | R5 | R5 | R5 | X4 | X4 | **83** | MZ736261 | R5 | X4 | X4 | R5 | R5 | X4 |
| **24** | MT489583 | R5 | R5 | R5 | R5 | R5 | R5 | **84** | MZ736260 | R5 | X4 | X4 | R5 | R5 | X4 |
| **25** | MT489582 | X4 | R5 | R5 | R5 | R5 | X4 | **85** | MZ736259 | R5 | X4 | X4 | R5 | R5 | X4 |
| **26** | MT489629 | R5 | R5 | R5 | X4 | R5 | X4 | **86** | MZ736156 | R5 | X4 | X4 | R5 | R5 | X4 |
| **27** | MZ736238 | X4 | R5 | X4 | X4 | X4 | X4 | **87** | MZ736155 | R5 | R5 | R5 | R5 | R5 | R5 |
| **28** | MZ736237 | X4 | R5 | R5 | R5 | X4 | X4 | **88** | MZ736258 | R5 | X4 | X4 | R5 | R5 | X4 |
| **29** | MT489581 | X4 | R5 | X4 | X4 | R5 | X4 | **89** | MZ736257 | R5 | X4 | X4 | R5 | R5 | X4 |
| **30** | MN387786 | X4 | R5 | R5 | R5 | R5 | X4 | **90** | MZ736154 | R5 | R5 | R5 | R5 | R5 | R5 |
| **31** | MN387783 | R5 | R5 | R5 | R5 | X4 | X4 | **91** | MZ736153 | R5 | R5 | R5 | R5 | R5 | R5 |
| **32** | MN387751 | R5 | R5 | X4 | R5 | R5 | X4 | **92** | MZ736256 | R5 | R5 | R5 | R5 | R5 | R5 |
| **33** | MN387749 | R5 | X4 | R5 | R5 | R5 | X4 | **93** | MZ736152 | R5 | R5 | R5 | R5 | R5 | R5 |
| **34** | MN387742 | R5 | R5 | R5 | R5 | R5 | R5 | **94** | MZ736151 | X4 | R5 | R5 | R5 | R5 | X4 |
| **35** | MN387741 | R5 | R5 | R5 | X4 | R5 | X4 | **95** | MZ736150 | R5 | R5 | R5 | R5 | R5 | R5 |
| **36** | MZ736283 | X4 | X4 | X4 | X4 | R5 | X4 | **96** | MZ736149 | R5 | R5 | R5 | R5 | R5 | R5 |
| **37** | MZ736236 | X4 | R5 | R5 | R5 | X4 | X4 | **97** | MZ736148 | X4 | R5 | R5 | R5 | R5 | X4 |
| **38** | MZ736282 | R5 | R5 | R5 | R5 | R5 | R5 | **98** | MZ736147 | X4 | R5 | R5 | R5 | R5 | X4 |
| **39** | MZ736235 | X4 | R5 | R5 | R5 | R5 | X4 | **99** | MT489640 | R5 | R5 | R5 | R5 | X4 | X4 |
| **40** | MZ736234 | X4 | R5 | R5 | R5 | X4 | X4 | **100** | MZ736255 | R5 | X4 | X4 | R5 | R5 | X4 |
| **41** | MZ736281 | R5 | X4 | R5 | R5 | R5 | X4 | **101** | MT489639 | X4 | R5 | X4 | X4 | R5 | X4 |
| **42** | MZ736280 | R5 | R5 | X4 | X4 | R5 | X4 | **102** | MT489638 | R5 | R5 | R5 | R5 | X4 | X4 |
| **43** | MZ736233 | X4 | R5 | R5 | R5 | X4 | X4 | **103** | MZ736254 | R5 | X4 | X4 | R5 | R5 | X4 |
| **44** | MZ736279 | R5 | R5 | R5 | R5 | R5 | R5 | **104** | MT489637 | X4 | R5 | R5 | R5 | R5 | X4 |
| **45** | MZ736278 | R5 | R5 | R5 | R5 | R5 | R5 | **105** | MZ736253 | R5 | X4 | X4 | R5 | R5 | X4 |
| **46** | MZ736232 | X4 | R5 | R5 | R5 | X4 | X4 | **106** | MT489636 | R5 | R5 | R5 | R5 | R5 | R5 |
| **47** | MZ736231 | X4 | R5 | R5 | R5 | R5 | X4 | **107** | MZ736252 | R5 | X4 | X4 | R5 | R5 | X4 |
| **48** | MZ736277 | R5 | R5 | R5 | R5 | R5 | R5 | **108** | MZ736251 | R5 | X4 | X4 | R5 | R5 | X4 |
| **49** | MZ736276 | R5 | R5 | R5 | R5 | R5 | R5 | **109** | MT489635 | R5 | R5 | R5 | R5 | R5 | R5 |
| **50** | MZ736230 | X4 | R5 | R5 | R5 | R5 | X4 | **110** | MT489630 | X4 | X4 | X4 | X4 | R5 | X4 |
| **51** | MZ736229 | X4 | R5 | R5 | R5 | R5 | X4 | **111** | MZ736250 | R5 | X4 | R5 | R5 | R5 | X4 |
| **52** | MZ736275 | R5 | R5 | R5 | R5 | R5 | R5 | **112** | MT489632 | X4 | R5 | R5 | R5 | R5 | X4 |
| **53** | MZ736274 | R5 | R5 | R5 | R5 | R5 | R5 | **113** | MT489631 | R5 | R5 | R5 | R5 | R5 | R5 |
| **54** | MZ736228 | X4 | R5 | R5 | R5 | R5 | X4 | **114** | MT489600 | X4 | R5 | X4 | R5 | R5 | X4 |
| **55** | MZ736273 | R5 | R5 | R5 | R5 | R5 | R5 | **115** | MZ736178 | X4 | R5 | R5 | R5 | R5 | X4 |
| **56** | MZ736227 | R5 | R5 | R5 | R5 | R5 | R5 | **116** | MZ736177 | X4 | R5 | R5 | R5 | R5 | X4 |
| **57** | MZ736226 | R5 | R5 | R5 | R5 | R5 | R5 | **117** | MZ736176 | X4 | R5 | R5 | R5 | R5 | X4 |
| **58** | MZ736184 | X4 | R5 | R5 | R5 | R5 | X4 | **118** | MT611503 | R5 | R5 | R5 | R5 | X4 | X4 |
| **59** | MZ736272 | R5 | R5 | R5 | R5 | R5 | R5 | **119** | MZ736249 | R5 | X4 | X4 | R5 | R5 | X4 |
| **60** | MZ736183 | X4 | R5 | R5 | R5 | R5 | X4 | **120** | MZ359100 | R5 | R5 | R5 | R5 | R5 | R5 |
|  | | **WebPSSM** | | **PhenoSeq** | | **Net charge** | | **Geno2Pheno** | | **11/25 rule** | | | **Final Result** | | |
| **Frequency** | | R5:68 (56.7%)  X4: 52 (43.3%) | | R5: 98 (81.7%)  X4: 22 (18.3%) | | R5: 90 (75%)  X4: 30 (25%) | | R5: 108 (90%)  X4: 12 (10%) | | R5: 18 (15%)  X4: 102 (85%) | | | R5: 41 (34.2%)  X4: 79 (65.8%) | | |

**Supplemental Table 1e:** Frequency of V3 tropism in subtype CRF35-AD.

| **#** | **Web PSSM** | **Pheno**  **Seq** | **Net charge** | **Geno2Pheno** | **11/25 rule** | **Final Result** | **#** | **Web PSSM** | **Pheno**  **Seq** | **Net charge** | **Geno2Pheno** | **11/25 rule** | **Final Result** |
| --- | --- | --- | --- | --- | --- | --- | --- | --- | --- | --- | --- | --- | --- |
| **1** | R5 | R5 | R5 | R5 | R5 | R5 | **63** | R5 | R5 | R5 | R5 | R5 | R5 |
| **2** | R5 | R5 | UD | R5 | R5 | R5 | **64** | R5 | R5 | R5 | R5 | R5 | R5 |
| **3** | R5 | R5 | R5 | R5 | R5 | R5 | **65** | R5 | R5 | UD | R5 | R5 | R5 |
| **4** | R5 | R5 | R5 | R5 | R5 | R5 | **66** | R5 | R5 | R5 | R5 | R5 | R5 |
| **5** | R5 | R5 | R5 | R5 | R5 | R5 | **67** | R5 | R5 | UD | R5 | R5 | R5 |
| **6** | X4 | R5 | X4 | X4 | X4 | X4 | **68** | R5 | R5 | R5 | R5 | R5 | R5 |
| **7** | R5 | R5 | R5 | R5 | R5 | R5 | **69** | R5 | R5 | R5 | R5 | R5 | R5 |
| **8** | R5 | X4 | R5 | X4 | R5 | X4 | **70** | R5 | R5 | R5 | R5 | R5 | R5 |
| **9** | R5 | R5 | R5 | R5 | R5 | R5 | **71** | R5 | R5 | UD | R5 | R5 | R5 |
| **10** | R5 | R5 | R5 | R5 | R5 | R5 | **72** | R5 | X4 | UD | R5 | R5 | X4 |
| **11** | R5 | R5 | R5 | R5 | R5 | R5 | **73** | R5 | X4 | R5 | R5 | R5 | X4 |
| **12** | R5 | R5 | R5 | R5 | R5 | R5 | **74** | R5 | R5 | R5 | R5 | R5 | R5 |
| **13** | R5 | R5 | R5 | R5 | R5 | R5 | **75** | R5 | R5 | R5 | R5 | R5 | R5 |
| **14** | R5 | R5 | R5 | R5 | R5 | R5 | **76** | R5 | R5 | R5 | R5 | R5 | R5 |
| **15** | R5 | R5 | R5 | R5 | R5 | R5 | **77** | R5 | R5 | UD | R5 | R5 | R5 |
| **16** | R5 | R5 | R5 | R5 | R5 | R5 | **78** | R5 | R5 | R5 | R5 | R5 | R5 |
| **17** | R5 | R5 | R5 | R5 | R5 | R5 | **79** | R5 | X4 | R5 | R5 | R5 | X4 |
| **18** | R5 | R5 | R5 | R5 | R5 | R5 | **80** | R5 | R5 | R5 | R5 | R5 | R5 |
| **19** | R5 | R5 | R5 | R5 | R5 | R5 | **81** | R5 | R5 | R5 | R5 | R5 | R5 |
| **20** | R5 | R5 | R5 | R5 | R5 | R5 | **82** | R5 | R5 | R5 | R5 | R5 | R5 |
| **21** | R5 | R5 | R5 | R5 | R5 | R5 | **83** | R5 | R5 | R5 | R5 | R5 | R5 |
| **22** | R5 | R5 | R5 | R5 | R5 | R5 | **84** | R5 | R5 | R5 | R5 | R5 | R5 |
| **23** | R5 | R5 | R5 | R5 | R5 | R5 | **85** | R5 | R5 | R5 | R5 | R5 | R5 |
| **24** | R5 | X4 | R5 | R5 | R5 | X4 | **86** | R5 | R5 | R5 | R5 | R5 | R5 |
| **25** | R5 | R5 | R5 | R5 | R5 | R5 | **87** | R5 | R5 | R5 | R5 | R5 | R5 |
| **26** | X4 | R5 | R5 | R5 | R5 | X4 | **88** | R5 | R5 | R5 | R5 | R5 | R5 |
| **27** | R5 | X4 | R5 | R5 | R5 | X4 | **89** | R5 | X4 | R5 | X4 | R5 | X4 |
| **28** | R5 | R5 | R5 | R5 | R5 | R5 | **90** | R5 | R5 | R5 | R5 | R5 | R5 |
| **29** | R5 | R5 | R5 | X4 | R5 | X4 | **91** | R5 | R5 | R5 | R5 | R5 | R5 |
| **30** | R5 | R5 | R5 | R5 | R5 | R5 | **92** | R5 | R5 | R5 | R5 | R5 | R5 |
| **31** | R5 | R5 | R5 | R5 | R5 | R5 | **93** | R5 | R5 | R5 | R5 | R5 | R5 |
| **32** | R5 | R5 | UD | R5 | R5 | R5 | **94** | R5 | R5 | R5 | R5 | R5 | R5 |
| **33** | R5 | X4 | UD | R5 | R5 | X4 | **95** | R5 | R5 | R5 | R5 | R5 | R5 |
| **34** | R5 | R5 | R5 | R5 | R5 | R5 | **96** | R5 | R5 | R5 | R5 | R5 | R5 |
| **35** | R5 | R5 | R5 | R5 | R5 | R5 | **97** | R5 | X4 | R5 | R5 | R5 | X4 |
| **36** | R5 | R5 | R5 | R5 | R5 | R5 | **98** | R5 | R5 | R5 | R5 | R5 | R5 |
| **37** | R5 | R5 | R5 | X4 | R5 | X4 | **99** | R5 | R5 | R5 | R5 | R5 | R5 |
| **38** | R5 | R5 | R5 | R5 | R5 | R5 | **100** | R5 | R5 | R5 | R5 | R5 | R5 |
| **39** | R5 | R5 | R5 | R5 | R5 | R5 | **101** | R5 | R5 | R5 | R5 | R5 | R5 |
| **40** | R5 | R5 | R5 | R5 | R5 | R5 | **102** | R5 | R5 | R5 | R5 | R5 | R5 |
| **41** | R5 | X4 | R5 | R5 | R5 | X4 | **103** | R5 | R5 | R5 | R5 | R5 | R5 |
| **42** | R5 | R5 | X4 | X4 | R5 | X4 | **104** | R5 | R5 | R5 | R5 | R5 | R5 |
| **43** | R5 | R5 | R5 | R5 | R5 | R5 | **105** | R5 | R5 | R5 | X4 | R5 | X4 |
| **44** | R5 | R5 | R5 | R5 | R5 | R5 | **106** | R5 | R5 | R5 | R5 | R5 | R5 |
| **45** | R5 | R5 | R5 | R5 | R5 | R5 | **107** | R5 | R5 | R5 | R5 | R5 | R5 |
| **46** | R5 | R5 | R5 | R5 | R5 | R5 | **108** | R5 | R5 | R5 | R5 | R5 | R5 |
| **47** | R5 | R5 | R5 | R5 | R5 | R5 | **109** | R5 | X4 | R5 | X4 | R5 | X4 |
| **48** | R5 | R5 | R5 | R5 | R5 | R5 | **110** | R5 | R5 | R5 | R5 | R5 | R5 |
| **49** | R5 | R5 | UD | R5 | R5 | R5 | **111** | R5 | R5 | R5 | R5 | R5 | R5 |
| **50** | R5 | R5 | R5 | R5 | R5 | R5 | **112** | R5 | R5 | R5 | X4 | R5 | X4 |
| **51** | R5 | R5 | R5 | R5 | R5 | R5 | **113** | R5 | R5 | R5 | R5 | R5 | R5 |
| **52** | R5 | R5 | R5 | R5 | R5 | R5 | **114** | R5 | R5 | R5 | R5 | R5 | R5 |
| **53** | R5 | R5 | R5 | R5 | R5 | R5 | **115** | R5 | R5 | R5 | R5 | R5 | R5 |
| **54** | R5 | R5 | R5 | R5 | R5 | R5 | **116** | R5 | R5 | R5 | R5 | R5 | R5 |
| **55** | R5 | R5 | R5 | R5 | R5 | R5 | **117** | R5 | R5 | R5 | R5 | R5 | R5 |
| **56** | R5 | R5 | UD | R5 | R5 | R5 | **118** | R5 | R5 | UD | R5 | R5 | R5 |
| **57** | R5 | R5 | R5 | R5 | R5 | R5 | **119** | R5 | X4 | UD | R5 | R5 | X4 |
| **58** | R5 | R5 | R5 | R5 | R5 | R5 | **120** | R5 | X4 | UD | X4 | R5 | X4 |
| **59** | R5 | R5 | R5 | R5 | R5 | R5 | **121** | R5 | R5 | R5 | R5 | R5 | R5 |
| **60** | R5 | R5 | R5 | R5 | R5 | R5 | **122** | R5 | R5 | R5 | R5 | R5 | R5 |
| **61** | R5 | R5 | R5 | R5 | R5 | R5 | **123** | R5 | R5 | R5 | R5 | R5 | R5 |
| **62** | R5 | R5 | R5 | R5 | R5 | R5 |  |  |  |  |  |  |  |
|  | **WebPSSM** | | **PhenoSeq** | | **Net charge** | | **Geno2Pheno** | | **11/25 rule** | | **Final Result** | | |
| **Frequency** | R5: 121 (98.4%)  X4: 2 (1.6%) | | R5:118 (89.4%)  X4:5 (10.6%) | | R5: 116 (87.8%)  X4: 2 (1.6%)  UD: 5 (10.6%) | | R5 (91.9%)  X4 (8.1%) | | R5 (99.2%)  X4 (0.8%) | | R5 (83.7%)  X4 (16.3%) | | |

**Supplementary Table 2:** Green and yellow boxes indicate the most and least tropisms, respectively, for each subtype (A (a), B (b), C (c), AE (d), and CRF35-AD (e)).

| **a** | **Subtype A** | Web PSSM | Geno2Pheno | PhenoSeq | Net charge | 11/25 rule |
| --- | --- | --- | --- | --- | --- | --- |
| Web PSSM | | - | R | R | R | R |
| Geno2Pheno | | 112 (93.3%) | - | 104 (86.6%) | 74 (61.1%) | 83 (69.2%) |
| PhenoSeq | | 106 (88.3%) | R | - | R | R |
| Net charge | | 77 (64.2%) | R | 72 (60%) | - | R |
| 11/25 rule | | 79 (65.8%) | R | 75 (62.5%) | 85 (70.83) | - |

| **b** | **Subtype B** | Web PSSM | Geno2Pheno | PhenoSeq | Net charge | 11/25 rule |
| --- | --- | --- | --- | --- | --- | --- |
| Web PSSM | | - | R | R | R | R |
| Geno2Pheno | | 93 (77.5%) | - | 54 (45%) | 87 (72.5%) | 102 (85%) |
| PhenoSeq | | 73 (60.8%) | R | - | R | R |
| Net charge | | 60 (50%) | R | 84 (70%) | - | R |
| 11/25 rule | | 98 (81.6%) | R | 66 (55%) | 50 (41.6%) | - |

| **c** | **Subtype C** | Web PSSM | Geno2Pheno | PhenoSeq | Net charge | 11/25 rule |
| --- | --- | --- | --- | --- | --- | --- |
| Web PSSM | | - | R | R | R | R |
| Geno2Pheno | | 111 (92.5%) | - | 107 (89.2%) | 84 (70%) | 117 (97.5%) |
| PhenoSeq | | 104 (86.6%) | R | - | R | R |
| Net charge | | 87 (72.5%) | R | 85 (70.8%) | - | R |
| 11/25 rule | | 112 (93.3%) | R | 110 (91.6%) | 85 (70.8%) | - |

| **d** | **Subtype AE** | Web PSSM | Geno2Pheno | PhenoSeq | Net charge | 11/25 rule |
| --- | --- | --- | --- | --- | --- | --- |
| Web PSSM | | - | R | R | R | R |
| Geno2Pheno | | 74 (61.1%) | - | 88 (73.3%) | 95 (79.2%) | 92 (76.6%) |
| PhenoSeq | | 74 (61.1%) | R | - | R | R |
| Net charge | | 57 (47.5%) | R | 103 (85.8%) | - | R |
| 11/25 rule | | 78 (65%) | R | 78 (65%) | 73 (60.8%) | - |

| **e** | **CRF35-AD** | Web PSSM | Geno2Pheno | PhenoSeq | Net charge | 11/25 rule |
| --- | --- | --- | --- | --- | --- | --- |
| Web PSSM | | - | R | R | R | R |
| Geno2Pheno | | 113 (91.9%) | - | 108 (87.8%) | 103 (83.7%) | 114(92.7%) |
| PhenoSeq | | 108 (87.8%) | R | - | R | R |
| Net charge | | 108 (87.8%) | R | 99 (80.5%) | - | R |
| 11/25 rule | | 122 (99.2%) | R | 109 (88.6%) | 109 (88.6%) | - |

**Supplemental Table 3:** The results of N-glycosylation site prediction, T8V, and crown motif of all subtypes based on V3 tropism results (A (a), B (b), C (c), and AE (d), and CRF35-AD (e)).

| **a /Subtype** | **V3 Tropism** | **N-glycosylation** | **Crown motif (GPGQ)** | **T8V Mutation** |
| --- | --- | --- | --- | --- |
| **A** | **Web PSSM** |  | | |
|  | R5:114 (95%)  X4: 6 (5%) | R5: 112/114 (98.3%)  X4: 4/6 (66.6%) | R5: 83/114 (72.8%)  X4: 5/6 (83.3%) | R5: 0/114 (0%)  X4: 0/6 (0%) |
|  | **PhenoSeq** |  | | |
|  | R5:107 (89.2%)  X4:13 (10.8%) | R5:106/107 (99.1%)  X4:10/13 (76.9%) | R5:82/107 (76.6%)  X4:6/13 (46.2%) | R5:0/107 (0%)  X4:0/13 (0%) |
|  | **Net charge** |  | | |
|  | R5: 73 (60.8%)  X4: 47 (39.2%) | R5: 71/73 (97.3%)  X4: 45/47 (95.7%) | R5: 63/73 (86.3%)  X4: 25/47 (53.2%) | R5: 0/73 (0%)  X4: 0/47 (0%) |
|  | **Geno2pheno** |  | | |
|  | R5: 120 (100%) | R5: 116/120 (96.6%) | R5: 88/120 (73.3%) | R5: 0/120 (0%) |
|  | **11/25 rule** |  | | |
|  | R5: 83 (69.2%)  X4:37 (30.8%) | R5: 80/83 (96.4%)  X4: 36/37 (97.3%) | R5: 67/83 (80.7%)  X4: 21/37 (56.7%) | R5: 0/83 (0%)  X4: 0/37 (0%) |
|  | **Final result** |  | | |
|  | R5: 57 (47.5%)  X4: 63 (52.5%) | R5: 57/57 (100%)  X4: 59/63 (93.6%) | R5: 29/57 (50.9%)  X4: 38/63 (60.3%) | R5: 0/57 (0%)  X4: 0/63 (0%) |

| **b /Subtype** | **V3 Tropism** | **N-glycosylation** | **Crown motif (GPGQ)** | **T8V Mutation** |
| --- | --- | --- | --- | --- |
| **B** | **Web PSSM** |  | | |
|  | R5: 100 (83.3%)  X4: 20 (16.7%) | R5: 98/100 (98%)  X4: 18/20 (90%) | R5: 0/100 (0%)  X4: 0/20 (0%) | R5: 0/100 (0%)  X4: 0/20 (0%) |
|  | **PhenoSeq** |  | | |
|  | R5: 53 (44.2%)  X4: 67 (55.8%) | R5:53/53 (100%)  X4:63/67 (94.1%) | R5:0/53 (0%)  X4:0/67 (0%) | R5:0/53 (0%)  X4:0/67 (0%) |
|  | **Net charge** |  | | |
|  | R5: 41 (34.2%)  X4: 79 (65.8%) | R5: 40/41 (97.6%)  X4: 76/79 (96.2%) | R5: 0/41 (0%)  X4: 0/79 (0%) | R5: 0/41 (0%)  X4: 0/79 (0%) |
|  | **Geno2pheno** |  | | |
|  | R5: 111 (92.5%)  X4: 9 (7.5%) | R5: 107/111(96.4%)  X4: 9/9 (100%) | R5: 0/111(0%)  X4: 0/9 (0%) | R5: 0/111(0%)  X4: 0/9 (0%) |
|  | **11/25 rule** |  | | |
|  | R5:104 (86.7%)  X4:16 (13.3%) | R5: 102/104 (98.1%)  X4: 14/16 (87.5%) | R5: 0/104 (0%)  X4: 0/16 (0%) | R5: 0/104 (0%)  X4: 0/16 (0%) |
|  | **Final result** |  | | |
|  | R5: 25 (20.8%)  X4: 95 (79.2%) | R5: 25/25 (100%)  X4: 91/95 (95.8%) | R5: 0/25 (0%)  X4: 0/95 (0%) | R5: 0/25 (0%)  X4: 0/95 (0%) |

| **c /Subtype** | **V3 Tropism** | **N-glycosylation** | **Crown motif (GPGQ)** | **T8V Mutation** |
| --- | --- | --- | --- | --- |
| **C** | **Web PSSM** |  | | |
|  | R5: 113 (94.2%)  X4: 7 (5.8%) | R5: 111/113 (98.3%)  X4: 6/7 (85.7%) | R5: 109/113 (96.5%)  X4: 6/7 (85.7%) | R5: 0/113 (0%)  X4: 0/7 (0%) |
|  | **PhenoSeq** |  | | |
|  | R5: 109 (90.8%)  X4: 11 (9.2%) | R5: 108/109 (99.1%)  X4: 9/11 (81.1%) | R5: 107/109 (98.2%)  X4: 8/11 (72.7%) | R5:0/109 (0%)  X4:0/11 (0%) |
|  | **Net charge** |  | | |
|  | R5: 85 (70.8%)  X4: 35 (29.2%) | R5: 83/85 (97.6%)  X4: 34/35 (97.2%) | R5: 84/85 (98.8%)  X4: 31/35 (88.6%) | R5: 0/85 (0%)  X4: 0/35 (0%) |
|  | **Geno2pheno** |  | | |
|  | R5: 118 (98.3%)  X4: 2 (1.7%) | R5: 115/118 (97.5%)  X4: 2/2 (100%) | R5: 113/118 (95.7%)  X4: 2/2 (100%) | R5: 0/118(0%)  X4: 0/2 (0%) |
|  | **11/25 rule** |  | | |
|  | R5: 119 (99.2%)  X4: 1 (0.8%) | R5: 116/119 (97.5%)  X4: 1/1 (100%) | R5: 114/119 (95.8%)  X4: 1/1 (100%) | R5: 0/119 (0%)  X4: 0/1 (0%) |
|  | **Final result** |  | | |
|  | R5: 78 (65%)  X4: 42 (35%) | R5: 78/78 (100%)  X4: 39/42 (92.8%) | R5: 78/78 (100%)  X4: 37/42 (88.1%) | R5: 0/78 (0%)  X4: 0/42 (0%) |

| **d /Subtype** | **V3 Tropism** | **N-glycosylation** | **Crown motif (GPGQ)** | **T8V Mutation** |
| --- | --- | --- | --- | --- |
| **CRF01-AE** | **Web PSSM** |  | | |
|  | R5: 68 (56.7%)  X4: 52 (43.3%) | R5: 66/68 (97.05%)  X4: 43/52 (82.7%) | R5: 46/68 (67.6%)  X4: 31/52 (59.6%) | R5: 0/68 (0%)  X4: 0/52 (0%) |
|  | **PhenoSeq** |  | | |
|  | R5: 98 (81.7%)  X4: 22 (18.3%) | R5: 90/98 (91.8%)  X4: 19/22 (86.4%) | R5: 75/98 (76.5%)  X4: 2/22 (9.1%) | R5:0/98 (0%)  X4:0/22 (0%) |
|  | **Net charge** |  | | |
|  | R5: 90 (75%)  X4: 30 (25%) | R5: 87/90 (96.6%)  X4: 22/30 (73.3%) | R5: 72/90 (80%)  X4: 5/30 (16.6%) | R5: 0/90 (0%)  X4: 0/30 (0%) |
|  | **Geno2pheno** |  | | |
|  | R5: 108 (90%)  X4: 12 (10%) | R5: 104/108 (96.3%)  X4: 5/12 (41.6%) | R5: 72/108 (66.6%)  X4: 5/12 (41.6%) | R5: 0/108(0%)  X4: 0/12 (0%) |
|  | **11/25 rule** |  | | |
|  | R5: 102 (85%)  X4: 18 (15%) | R5: 91/102 (89.2%)  X4: 18/18 (100%) | R5: 59/102 (57.8%)  X4: 18/18 (100%) | R5: 0/102 (0%)  X4: 0/18 (0%) |
|  | **Final result** |  | | |
|  | R5: 41 (34.2%)  X4: 79 (65.8%) | R5: 41/102 (40.2%)  X4: 68/79 (86.1%) | R5: 39/102 (38.3%)  X4: 38/79 (48.1%) | R5: 0/41 (0%)  X4: 0/79 (0%) |

| **e / Subtype** | **V3 Tropism** | **N-glycosylation** | **Crown motif (GPGQ)** | **T8V Mutation** |
| --- | --- | --- | --- | --- |
| **CRF35-AD** | **Web PSSM** |  |  |  |
|  | R5: 121 (98.4%)  X4: 2 (1.6%) | R5: 118/121 (97.5%)  X4: 2/2 (100%) | R5: 117/121 (96.7%)  X4: 2/2 (100%) | R5: 0/121 (0%)  X4: 0/2 (0%) |
|  | **PhenoSeq** |  | | |
|  | R5:110 (89.4%)  X4:13 (10.6%) | R5:107/110 (97.3%)  X4:13/13 (100%) | R5:106/110 (96.4%)  X4:13/13 (100%) | R5:0/118 (0%)  X4:0/5 (0%) |
|  | **Net charge** |  | | |
|  | R5: 108 (87.8%)  X4: 2 (1.6%)  UD: 13 (10.6%) | R5: 106/108 (98.1%)  X4: 1/2 (50%)  UD: 13/13 (100%) | R5: 104/108 (96.3%)  X4: 2/2 (100%)  UD: 13/13 (100%) | R5: 0/108 (0%)  X4: 0/2 (0%)  UD: 0/10 (0%) |
|  | **Geno2pheno** |  | | |
|  | R5: 113(91.9%)  X4: 10 (8.1%) | R5: 111/113(98.2%)  X4: 9/10 (90%) | R5: 109/113(96.5%)  X4: 10/10 (100%) | R5: 0/113(0%)  X4: 0/10 (0%) |
|  | **11/25 rule** |  | | |
|  | R5: 122 (99.2%)  X4: 1 (0.8%) | R5: 119/122 (97.5%)  X4: 1/1 (100%) | R5: 118/122 (96.7%)  X4: 1/1 (100%) | R5: 0/122 (0%)  X4: 0/1 (0%) |
|  | **Final result** |  | | |
|  | R5: 103 (83.7%)  X4: 20 (16.3%) | R5: 101/103 (98.1%)  X4: 19/20 (95%) | R5: 102/103 (99.1%)  X4: 17/20 (85%) | R5: 0/103 (0%)  X4: 0/20 (0%) |
